# Fluorine-Modulated MXene-Derived Catalysts for Multiphase Sulfur Conversion in Lithium–Sulfur Battery

**DOI:** 10.1007/s40820-024-01482-6

**Published:** 2024-08-12

**Authors:** Qinhua Gu, Yiqi Cao, Junnan Chen, Yujie Qi, Zhaofeng Zhai, Ming Lu, Nan Huang, Bingsen Zhang

**Affiliations:** 1 Shenyang National Laboratory for Materials Science, Institute of Metal Research, Chinese Academy of Sciences, Shenyang, 110016 People’s Republic of China; 2 School of Materials Science and Engineering, University of Science and Technology of China, Shenyang, 110016 People’s Republic of China; 3 The Joint Laboratory of MXene Materials, Key Laboratory of Functional Materials Physics and Chemistry of the Ministry of Education, Key Laboratory of Preparation and Application of Environmental Friendly Materials of the Ministry of Education, Jilin Normal University, Changchun, 130103 People’s Republic of China

**Keywords:** Catalysis, Fluorination, MXene, Lithium–sulfur battery, Shuttle effect

## Abstract

Fluorine owing to its inherently high electronegativity exhibits charge delocalization and ion dissociation capabilities; as a result, there has been an influx of research studies focused on the utilization of fluorides to optimize solid electrolyte interfaces and provide dynamic protection of electrodes to regulate the reaction and function performance of batteries. Nonetheless, the shuttle effect and the sluggish redox reaction kinetics emphasize the potential bottlenecks of lithium–sulfur batteries. Whether fluorine modulation regulate the reaction process of Li–S chemistry? Here, the TiOF/Ti_3_C_2_ MXene nanoribbons with a tailored F distribution were constructed via an NH_4_F fluorinated method. Relying on in situ characterizations and electrochemical analysis, the F activates the catalysis function of Ti metal atoms in the consecutive redox reaction. The positive charge of Ti metal sites is increased due to the formation of O–Ti–F bonds based on the Lewis acid–base mechanism, which contributes to the adsorption of polysulfides, provides more nucleation sites and promotes the cleavage of S–S bonds. This facilitates the deposition of Li_2_S at lower overpotentials. Additionally, fluorine has the capacity to capture electrons originating from Li_2_S dissolution due to charge compensation mechanisms. The fluorine modulation strategy holds the promise of guiding the construction of fluorine-based catalysts and facilitating the seamless integration of multiple consecutive heterogeneous catalytic processes.

**Supplementary Information:**

The online version contains supplementary material available at 10.1007/s40820-024-01482-6.

## Highlights

- By introducing fluorine modulation into MXene, a new MXene-derived material TiOF/Ti_3_C_2_ was successfully synthesized with a distinctive three-dimensional structure and a tailored F distribution.
- In situ characterizations and electrochemical analyses demonstrate that TiOF/Ti_3_C_2_ catalysts effectively coupled the multiphase sulfur species conversion processes.
- The investigations reveal that the theoretical basis of the fluorine catalysis in Li–S batteries originated from Lewis acid–base mechanisms and charge compensation mechanisms.

**Supplementary Information:**

The online version contains supplementary material available at 10.1007/s40820-024-01482-6.

## Graphical abstract

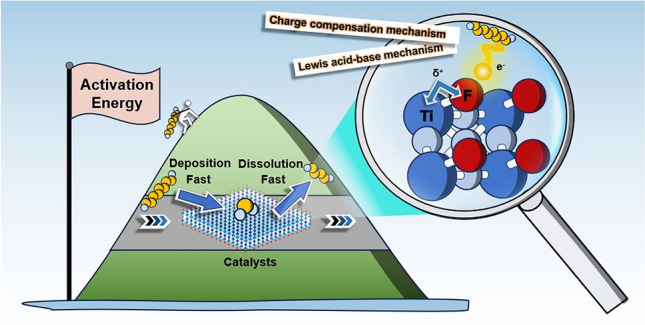

## Introduction

The redox kinetics and shuttle effect are responsible for the bottlenecks of a critical application for lithium–sulfur (Li–S) batteries. How to accelerate sulfur conversion and reduce the accumulation of lithium polysulfides (LiPSs) is crucial in regulating the Li–S reaction processes [1, 2]. When reacting with Li^+^, sulfur species undergo a solid-liquid phase transformation to form Li_2_S_4_, followed by a liquid-solid phase transformation to form Li_2_S. Considering the evolution of sulfur species undergoes a series of adsorption–conversion–desorption processes, there will be a considerable need for the systematic design and development of catalysts to drive phase transformation processes [3, 4]. Transition metal compounds [5–10], metals [11–14], metal-free materials [15, 16], heterostructures [17, 18], etc., provide a practical roadmap to catalysts in Li–S batteries. Through direct comparison of the catalysis-relevant metrics that guide the regulation of reaction processes in the independent reaction step, the design of multi-function catalysts and the understanding of the catalytic effect in the dependent redox reaction of Li–S batteries can promote a greater utilization of catalysis.

Fluorine, with a small atomic radius and high electronegativity, stands as the most diminutive electron-withdrawing group [19–21]. Owing to these distinctive properties, fluorine modulation engineering is regarded as a potent method for customizing material characteristics with precision. In the realm of catalysis, the judicious employment of fluorinating agents, such as NH_4_F, facilitates the enrichment of catalyst surfaces with fluorine, leading to unique morphologies, enhanced catalytic activity, and remarkable stability [22, 23]. In energy storage, fluorine modulation is widely used in electrode materials and electrolytes for various battery types, including lithium-ion, lithium-metal, potassium-ion, and sodium-ion batteries [24–30]. The advantages of fluorine are manifold: Firstly, it introduces metal-F ligands that expand the electrochemical working window, which is critical for energy storage devices. Secondly, it can protect the electrode structure. For example, through the interface engineering strategy, the conversion reaction of metal fluorides is utilized to generate the LiF passivation and alloy layers. This metal fluoride-modified lithium anodes not only suppress undesirable side reactions and the growth of lithium dendrites [31]. Thirdly, metal fluorides can facilitate the lithium ion transportation during the redox reaction of sulfur species [32]. Lastly, the formation of metal-F bonds stabilizes electrode structures, ensuring long-term performance [33–35]. Whether fluorine modulation regulates the reaction process of Li–S chemistry? The exact mechanisms by which fluorine influences electron distribution and controls active site behavior in catalysts require more profound investigation.

Herein, MXene serves as a host material with excellent conductivity and chemical adsorption sites, enhancing the ability to physically and chemically adsorb polysulfides and facilitate redox reactions. Based on MXene materials as the research foundation, this work comprehensively investigates fluorine modulation engineering to promote the coupled conversion of multiphase sulfur. Employing a two-step hydrothermal method, we synthesized three-dimensional TiOF/Ti_3_C_2_ catalysts through the in situ derivatization of Ti_3_C_2_ nanosheets. The unique catalyst significantly enhances the interaction between metal sites and polysulfides. In situ characterizations laid bare the substantial influence on electron/ion transport. The moderate interaction aids the cleavage of S–S bonds and the decomposition of Li–S bonds during the redox reaction, respectively. Owing to the mechanism of Lewis acid–base and charge compensation, the nucleation and decomposition barriers of Li_2_S were notably diminished. This study substantiates the feasibility of achieving efficient coupling of multiphase reactions throughout the discharge and charge processes, shedding light on the intricate catalytic mechanisms in Li–S batteries.

## Experimental Section

### Material Synthesis

Synthesis of TiOF/Ti_3_C_2_ and TiO/Ti_3_C_2_: MXene solution was prepared as our previous report [36]. Initially, 5 mg mL^−1^ Ti_3_C_2_ MXene solution was mixed with 4 M NaOH (99.9%, Sinopharm Chemical Reagent Co., Ltd.) solution under vigorous magnetic stirring for 24 h. After the reaction, the TiO/Ti_3_C_2_ powders were collected by filter and rinsing to remove residual Na^+^ and obtained after drying in a freeze dryer. 200 mg NH_4_F (99.9%, Alfa Aesar Chemical Co., Ltd.) was dispersed in 50 mL deionized water using a sonication treatment and was then transferred into a 100 mL autoclave at 170 °C for 4 h. TiO/Ti_3_C_2_ powder was dispersed in NH_4_F solution after pyrolysis, ultrasonic for 1 h, and then reacted at 60 °C for 2 h. The resultant TiOF/Ti_3_C_2_ powders were collected by filter and rinsing to remove residual and after drying in a freeze dryer.

Synthesis of TiOF/Ti_3_C_2_/S cathodes: The TiOF/Ti_3_C_2_/S cathodes were fabricated by a melt-diffusion method. 30 wt% of TiOF/Ti_3_C_2_ powders were mixed with 70 wt% of sulfur by milling in a mortar. Then, the mixture was transferred into an autoclave filled with Argon and then co-heated at 155 °C for 12 h. Afterward, TiOF/Ti_3_C_2_/S composites were collected. For comparison, TiO/Ti_3_C_2_/S and Ti_3_C_2_/S were also prepared by a similar method.

### Cell Assembly and Electrochemical Measurements

#### Lithium Polysulfide Adsorption Tests

To prepare the Li_2_S_6_ electrolyte, Li_2_S and S with a molar ratio of 1: 5 were added to a 1: 1 (v/v) DOL/DME mixture and stirred overnight at 50 °C in a glove box. The concentration of the obtained Li_2_S_6_ electrolyte was 0.2 M. 20 mg of TiOF/Ti_3_C_2_, TiO/Ti_3_C_2_ or Ti_3_C_2_ powders as the adsorbent was soaked into 4 mL of the as-prepared Li_2_S_6_ solution, respectively. Digital images were taken before and after resting.

#### Assembly of Symmetric Batteries

The preparation process of electrodes for symmetrical batteries does not contain sulfur melt-diffusion. The TiOF/Ti_3_C_2_, TiO/Ti_3_C_2_ or Ti_3_C_2_ powders were dispersed in N-methyl-2-pyrrolidone (NMP, AR, Future MaterialsTechnology Co., Ltd.) at a weight ratio of 3:1 with stirring and then the slurry was dropped onto the carbon paper which was cut into circular pellets with a diameter of 10 mm after drying for use on the electrode disks with a diameter of 10 mm. Those disks were used as identical working and counter electrodes, while 40 μL Li_2_S_6_ electrolyte was added to each battery.

#### Measurement for the Li_2_S Deposition and Dissolution

The TiOF/Ti_3_C_2_, TiO/Ti_3_C_2_ and Ti_3_C_2_ powders were dispersed in NMP with stirring, respectively. The slurry was loaded onto carbon paper with a diameter of 10 mm were used as cathodes and lithium foils were used as anodes. To prepare Li_2_S_8_ electrolyte, Li_2_S and sulfur powders with a molar ratio of 1:7 were added to tetraglyme and stirred at 50 °C for 24 h. A 0.2 mol L^−1^ of Li_2_S_8_ solution was obtained to be used as the electrolyte. During the preparation of Li–S batteries, 20 µL Li_2_S_8_ electrolyte was used as the catholyte while 20 µL of control electrolyte without Li_2_S_8_ was used as the anolyte. The assembled batteries were first discharged galvanostatically under a current of 0.112 mA until the voltage decreased to 2.06 V and then discharged potentiostatically at 2.05 V for Li_2_S deposition. For the Li_2_S dissolution test, the prepared batteries were first galvanostatically discharged at a current of 0.1 mA to 1.7 V, followed by potentiostatically charged under 2.42 V.

#### Li–S Batteries Assembly and Electrochemical Measurements

The electrodes were fabricated by mixing active materials (TiOF/Ti_3_C_2_, TiO/Ti_3_C_2_ or Ti_3_C_2_), Super-P, and PVDF binder with a weight ratio of 7:2:1 in NMP, which was then coated onto an Al foil and vacuum-dried at 55 °C for 12 h. After vacuum-drying, the Al foils were cut into circular disks with diameters of 10 mm to serve as cathodes. The sulfur loading of a cathode was about 1.5 mg cm^−1^. CR2032 coin batteries (Canrd Technology Co. Ltd.) were assembled in an Ar-filled glovebox with the concentration of moisture and oxygen below 0.5 ppm, using lithium foils as the counter cathodes. Each battery was injected with 24 μL electrolyte solutions. The electrolyte solution consisting of 1.0 M lithium bis -trifluoromethanesulfonimide (LiTFSI) in 1:1 (v/v) DME: DOL and 1 wt% LiNO_3_. For the Li–S batteries with a high loading of sulfur, the electrolyte addition was loaded with the E/S ratio of 8:1. Specially, Li foil with a thickness of 50 μm was used to assemble battery with TiOF/Ti_3_C_2_ cathode in a large areal pouch cell. All batteries were aged for several hours before cycling to ensure adequate penetration of the electrolyte into the electrode. The electrochemical performances were measured using a NEWARE-CT4008 instrument within a voltage window of 1.7–2.8 V versus Li/Li^+^ at room temperature. The specific capacities were calculated based on the sulfur mass.

### Characterization Methods

The structure and morphology of Ti_2_C nanosheets and Co/Ti_2_C catalysts were characterized by an X-ray diffractometer operating (Karlsruhe, Germany) at 40 kV, 30 mA with Cu-*K* radiation (*λ* = 0.15405 nm) and field emission SEM (FEI Nova Nano 450) as well as an FEI Tecnai G^2^ F20 microscope equipped with HAADF-STEM and EDX detectors. X-ray photoelectron spectroscopy (XPS) characterizations were obtained by an ESCALAB 250 instrument (Waltham, United States) with Al Kα X-rays (1489.6 eV, 150 W, 50.0 eV pass energy). In situ Raman characterizations were conducted by Raman microscope (Horiba, Labram HR Evolution). In situ X-ray diffraction (XRD) was performed by Bruker, D8 X-ray diffraction; the operando cell device is purchased from Beijing Scistar Technology Co. Ltd.

## Results and Discussion

### Catalysts Design and Structural Characterizations

**Fig. 1:**
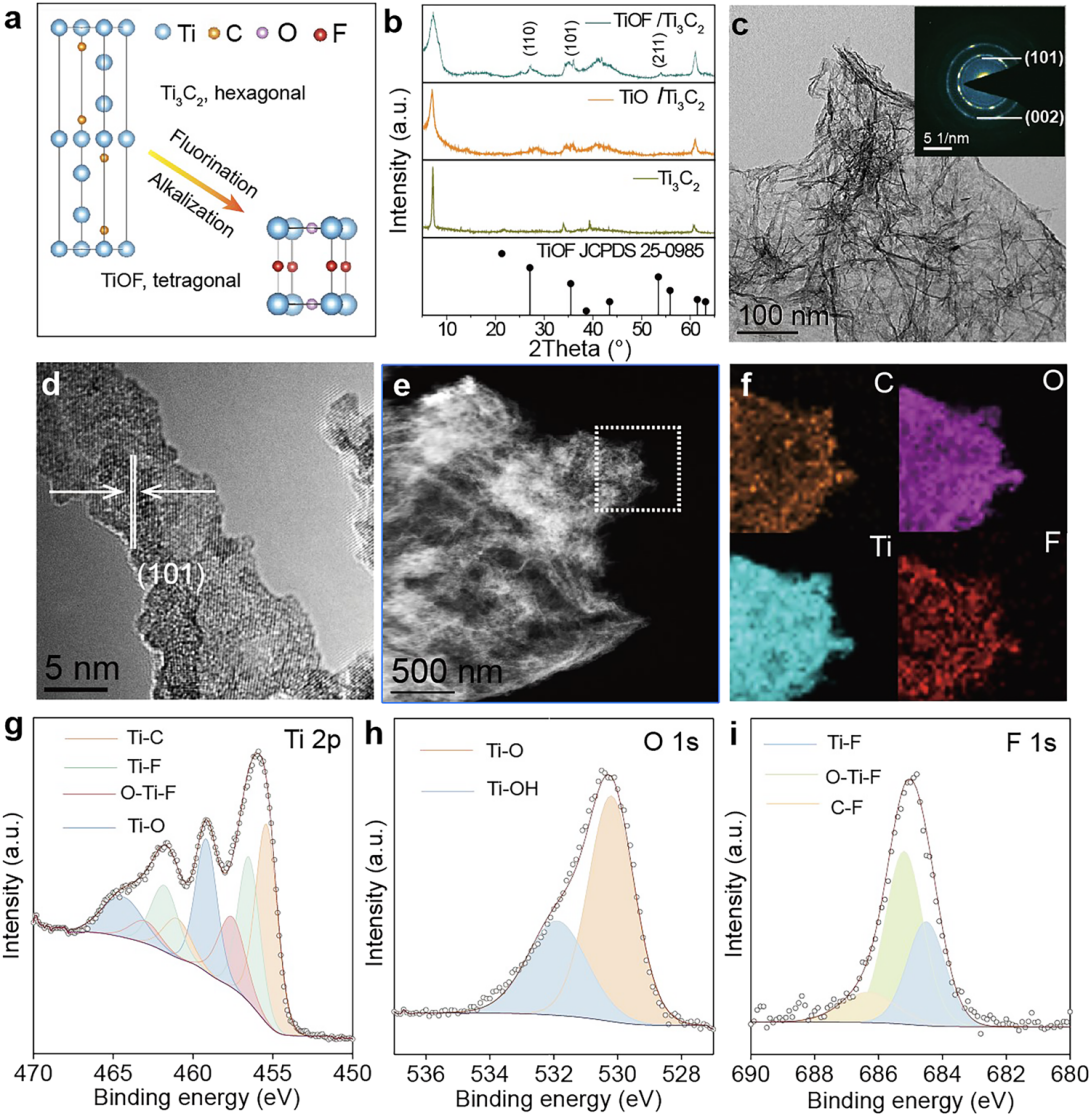
Structural and morphological characterizations of TiOF/Ti_3_C_2_ catalysts. **a** Unit cells of Ti_3_C_2_ and TiOF, represented in arbitrary units (a.u.).** b** XRD patterns of Ti_3_C_2_, TiO/Ti_3_C_2_ and TiOF/Ti_3_C_2_. **c** TEM image (the inset is the corresponding SAED) of TiOF/Ti_3_C_2_. **d** HRTEM image, **e** HAADF-STEM image and **f** the corresponding EDX elemental maps of TiOF/Ti_3_C_2_. XPS spectra of **g** Ti 2*p*, **h** O 1*s* and **i** F 1*s* for TiOF/Ti_3_C_2_ catalysts

The inherent versatility of MXene facilitates the synthesis of TiOF/Ti_3_C_2_ catalysts from Ti_3_C_2_ MXene through a two-step hydrothermal method (Fig. 1a). Initially, the MAX phase of Ti_3_AlC_2_ precursor was selectively etched the aluminum (Al) layers to obtain multilayered Ti_3_C_2_ MXene using hydrofluoric acid (HF) (Fig. S1) [36–39]. To increase the physical space for the electrode reaction of S, ultrasonic processing was applied to the multiple layers of MXene [40–43]. The process transformed morphologies from accordion-like to transparent and ultrathin two-dimensional (2D) structures (Fig. S2a). Following this, an alkalization treatment with NaOH solution was introduced, prompting the transformation of Ti_3_C_2_ nanosheets into TiO/Ti_3_C_2_ [44]. Transmission electron microscopy (TEM) images revealed an interwoven nanoribbon-nanosheet structure, displayed in Fig. S2b. Additionally, high-resolution (HR) TEM images, along with the corresponding FFTs, verified the formation of TiO nanoribbons due to the surface oxidation in air (Fig. S2c). Finally, using NH_4_F as the fluorine source after pyrolysis, a fluorination process was induced by F^−^ to obtain the TiOF/Ti_3_C_2_ catalysts.

The morphologies and structures of TiOF/Ti_3_C_2_ were examined by XRD, electron microscopy (EM) and XPS characterizations. The XRD patterns illustrate the transformation of Ti_3_C_2_ nanosheets into TiO through alkalization, ultimately yielding the TiOF structure as influenced by F^−^ (Fig. 1b). Characteristic diffraction peaks (110), (101), and (211) located at 27.1°, 35.5°, and 53.5° are attributed to TiOF. A shift to the left of the peak (002) and a marginal increase in the full width at half peak compared to pristine Ti_3_C_2_ nanosheets suggest an expansion in the layer spacing of MXene during fluorination, evidenced by the formation of TiOF nanoribbons. SEM and TEM images of TiOF/Ti_3_C_2_ reveal the preservation of the 3D structure, while high-angle annular dark-field scanning TEM (HAADF-STEM) and energy-dispersive X-ray spectroscopy (EDX) elemental maps illustrate the distribution of C, O, Ti, and F elements on the TiOF/Ti_3_C_2_ catalysts (Fig. 1e, f). This analysis suggests that TiOF nanoribbons are uniformly derived from the Ti_3_C_2_ parent (Figs. 1c and S1d). SAED and HRTEM also confirm that the formation of the TiOF phase is partially derived from Ti_3_C_2_ nanosheets (Fig. 1c, d). Delving into the chemical composition and electronic environment with post-fluorine modulation, XPS and electron energy loss spectroscopy (EELS) analyses were conducted (Figs. S3–S5). As shown in Table S1, the element content of TiOF/Ti_3_C_2_, TiO/Ti_3_C_2_, and Ti_3_C_2_ was characterized by XPS. The Ti 2*p* XPS spectrum exhibits peaks at 455.4, 456.6, 457.7, and 459.2 eV, correlating to Ti–C, Ti–F, O–Ti–F, and Ti–O bonds, respectively (Fig. 1g) [45]. A comparison of the Ti 2*p* XPS spectra of the TiO/Ti_3_C_2_ and Ti_3_C_2_ XPS spectrum reveals that the F element incorporation into the crystal lattice and induced partial phase transformation of Ti_3_C_2_ MXene, as evidenced by the presence of O-Ti-F bonds [46, 47]. This inference is supported by the F 1*s* XPS spectra and deconvolution analysis (Figs. 1i and S4), with peaks at 684.5, 685.2, and 686.4 eV designated to Ti–F, O–Ti–F, and C–F bonds, respectively. The formation of Ti–F and C–F bonds results from HF treatment, while O–Ti–F bonds signify the presence of TiOF. The O 1*s* XPS spectra display two peaks at 530.2 and 531.9 eV denoted Ti–O and Ti–OH (Fig. 1h) [48]. EELS analysis indicates a lower valence state of Ti in TiOF/Ti_3_C_2_ catalysts compared to TiO_2_, primarily due to the induction of F^−^ (Fig. S5) [49].

In general, F^−^ induction prompts MXene to incorporate fluorine into the crystal lattice, leading to a partial phase transformation in Ti_3_C_2_ MXene and the formation of the TiOF/Ti_3_C_2_ catalyst. This process not only maintains the 3D morphology but also sustains the TiOF crystalline phase derived from Ti_3_C_2_, optimizing MXene atomic arrangement.

### Analysis of Successive Heterogeneous Catalytic Processes

The Li–S chemistry is a typical redox process involving complex interactions between sulfur species and catalysts, including adsorption, reaction, and desorption [50]. To gain a profound insight into the catalytic effects and mechanisms of TiOF/Ti_3_C_2_ catalysts in continuous redox reactions, a series of electrochemical, visualization studies and in situ characterization analysis methods have been employed. These approaches are designed to explore the catalytic activity and kinetic behavior of the catalyst throughout discharge and charge processes.

#### Bifunctional Catalytic Activity Analysis

**Fig. 2:**
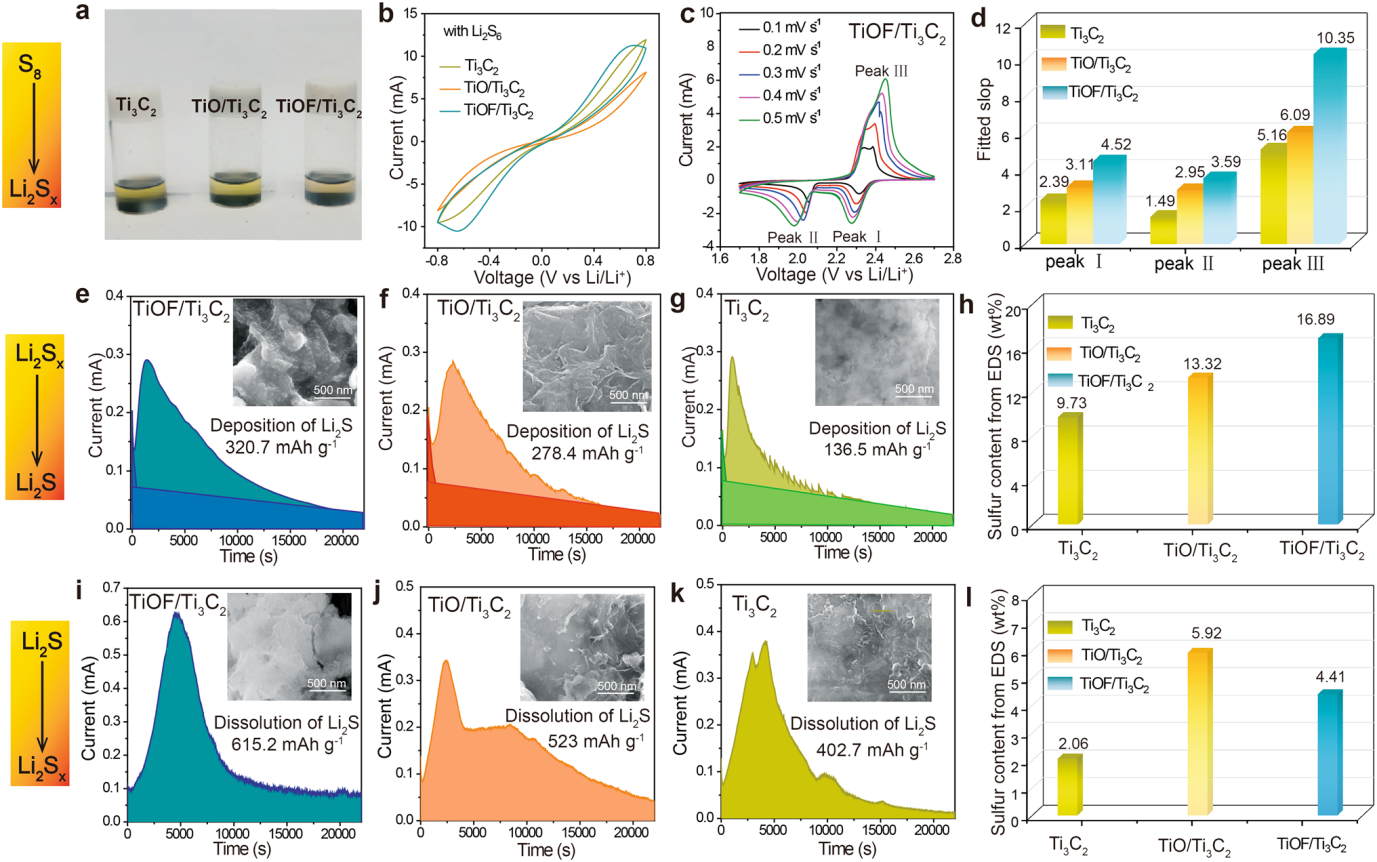
Bifunctional catalytic activity analysis. **a** Visualized adsorption of Li_2_S_6_ by TiOF/Ti_3_C_2_, TiO/Ti_3_C_2_ and Ti_3_C_2_. **b** CV curves of Li_2_S_6_ in symmetrical Li–S batteries at a scan rate of 5 mV s^−1^. **c** CV curves for TiOF/Ti_3_C_2_ batteries at different scan rates and **d** the corresponding linear fitting numerical statistics. Potentiostatic discharge curves of the Li_2_S deposition tests using **e** TiOF/Ti_3_C_2_, **f** TiO/Ti_3_C_2_ and **g** Ti_3_C_2_ as substrates (the insets are SEM images of the Li_2_S deposition morphology on the different substrates). **h** Statistics of sulfur content on different substrates after deposition of Li_2_S. Potentiostatic charge curves of the Li_2_S dissolution tests using **i** TiOF/Ti_3_C_2_, **g** TiO/Ti_3_C_2_ and **k** Ti_3_C_2_ as substrates (the insets are SEM images of the Li_2_S dissolution morphology on the different substrates). **l** Statistics of sulfur content on different substrates after dissolution of Li_2_S

*Chemisorption behavior* The rapid accumulation of LiPSs on the catalyst surface plays a crucial role in facilitating interactions with catalytic active sites. To evaluate the adsorption capabilities of TiOF/Ti_3_C_2_, TiO/Ti_3_C_2_, and Ti_3_C_2_, visual adsorption experiments were conducted using Li_2_S_6_ at a fixed concentration and volume as a model adsorbate for polysulfides, employing equal masses of the three catalyst powders as adsorbents. Figure 2a illustrates the results of the experiments. Notably, after a 0.5 h soaking period, the solution containing TiOF/Ti_3_C_2_ turned colorless, compared to the color of solutions containing TiO/Ti_3_C_2_ and Ti_3_C_2_ powders. It is suggested that the catalysts featuring the distribution of F elements establish a moderate interaction with LiPSs, which is a crucial prerequisite for the subsequent redox reaction.

*Solid–liquid reaction* In traditional Li–S batteries, the fundamental reaction mechanisms involve complex solid–liquid phase transitions that leading electron emission, thereby generating electrical energy [51]. During the discharge process, cyclo-octasulfur (S_8_) undergoes reduction, reacting with Li^+^ released from the Li anode. These reaction results in the formation of a series of intermediates [52]. It is worth noting that an excessive accumulation of LiPSs in the electrolyte can consume active species and hinder the transport of Li^+^. To evaluate the electrocatalytic activity of different materials, cyclic voltammetry (CV) curves of Li_2_S_6_ symmetric cells were acquired (Fig. 2b). The TiOF/Ti_3_C_2_ electrode exhibited both the largest peak area and highest peak current response, indicating the capability to accelerate the conversion of long-chain LiPSs to short-chain LiPSs. Moreover, CV curves of different cathodes at incremental scanning rates are performed (Figs. 2c and S6). During the cathodic scanning process, two reduction peaks were observed. The first peak (Peak I) can be attributed to the reduction of S_8_ into soluble LiPSs, followed by the second peak (Peak II), which represents the subsequent reduction of LiPSs into solid Li_2_S_2_/Li_2_S [53]. In the reversible anodic scan, Li_2_S_2_/Li_2_S is oxidized to LiPSs and finally to S_8_ (Peak III). Significantly, as the scan rate increasing, the TiOF/Ti_3_C_2_ cathode maintains well-defined CV curves and sharp redox peaks. According to the Randles–Sevcik equation, I = 2.686 × 10^5^ *n*^1.5^AD_Li_ + Cv^0.5^, it is evident that the peak current I exhibits a strong positive correlation with the square root of scan rate v [54]. Notably, the slopes of I-v^0.5^ for the TiOF/Ti_3_C_2_ cathode are greater than those of TiO/Ti_3_C_2_ and Ti_3_C_2_, demonstrating faster Li^+^ diffusion (Figs. 2d and S7). The results show that regulation F elements can significantly enhance the interface interaction between the electrode and polysulfides, thereby, facilitating the solid–liquid phase transition process and optimizing the diffusion efficiency of Li^+^.

*Liquid–solid reaction* During the discharge process, the liquid–solid process can contribute approximately three-quarters of the theoretical capacity, meaning that the facilitating phase transition process is significant to electrochemical performance [55]. As shown in Fig. 2e–g, the results of Li_2_S precipitation experiments reveal that the TiOF/Ti_3_C_2_ cathode exhibits the highest precipitation capacity of 320.7 mAh g^−1^. The result implies a higher catalytic efficiency in the precipitation of Li_2_S compared with those of TiO/Ti_3_C_2_ (278.4 mAh g^−1^) and Ti_3_C_2_ (136.5 mAh g^−1^) cathodes, according to Faraday’s law [56] (Figs. 2h and S8). Essentially, with the regulation of fluorine elements, TiOF/Ti_3_C_2_ can provide an increased number of nucleation sites for Li_2_S, accelerating nucleation rates, lowering nucleation energy barriers, and thereby enhancing battery performance.

*Solid–liquid reaction* The charging process of Li–S batteries is the reverse reaction of the discharging, also involving a phase transition process that requires overcoming significant energy barriers [57]. If the generated Li_2_S cannot be efficiently decomposed, it can lead to the accumulation of dead sulfur on the active sites of the catalyst, thereby compromising the electrochemical performance. Therefore, the Li_2_S dissolution process was further studied through potentiostatic measurements. As shown in Fig. 2i–k, the TiOF/Ti_3_C_2_ cathode demonstrates the highest decomposition capacity at 615.2 mAh g^−1^. This suggests a superior catalytic efficiency compared to the TiO/Ti_3_C_2_ (523 mAh g^−1^) and Ti_3_C_2_ (402.7 mAh g^−1^) cathodes. SEM and EDX results indicate that the surface morphology of TiOF/Ti_3_C_2_ electrode becomes smoother due to the dissolution of Li_2_S, according to the reduced sulfur content. (Figs. 2l and S9). This transformation demonstrated that the fluorine modulation optimizes the interactions at the three-phase interface, accelerating electron and ion transport across the electrode interfaces and thereby facilitating the Li_2_S decomposition.

Fluorine modification of the catalyst assumes a crucial role throughout the sulfur species phase reaction. The F modulation effectively homogenizes active reaction sites, thereby enhancing the transfer of ions and electrons at the three-phase interface of sulfur species to meet the catalytic electrode. As a result, the electrocatalytic effect enhances battery performance, improving both discharge and charge processes.

#### Reaction Kinetics and In Situ Characterizations

**Fig. 3:**
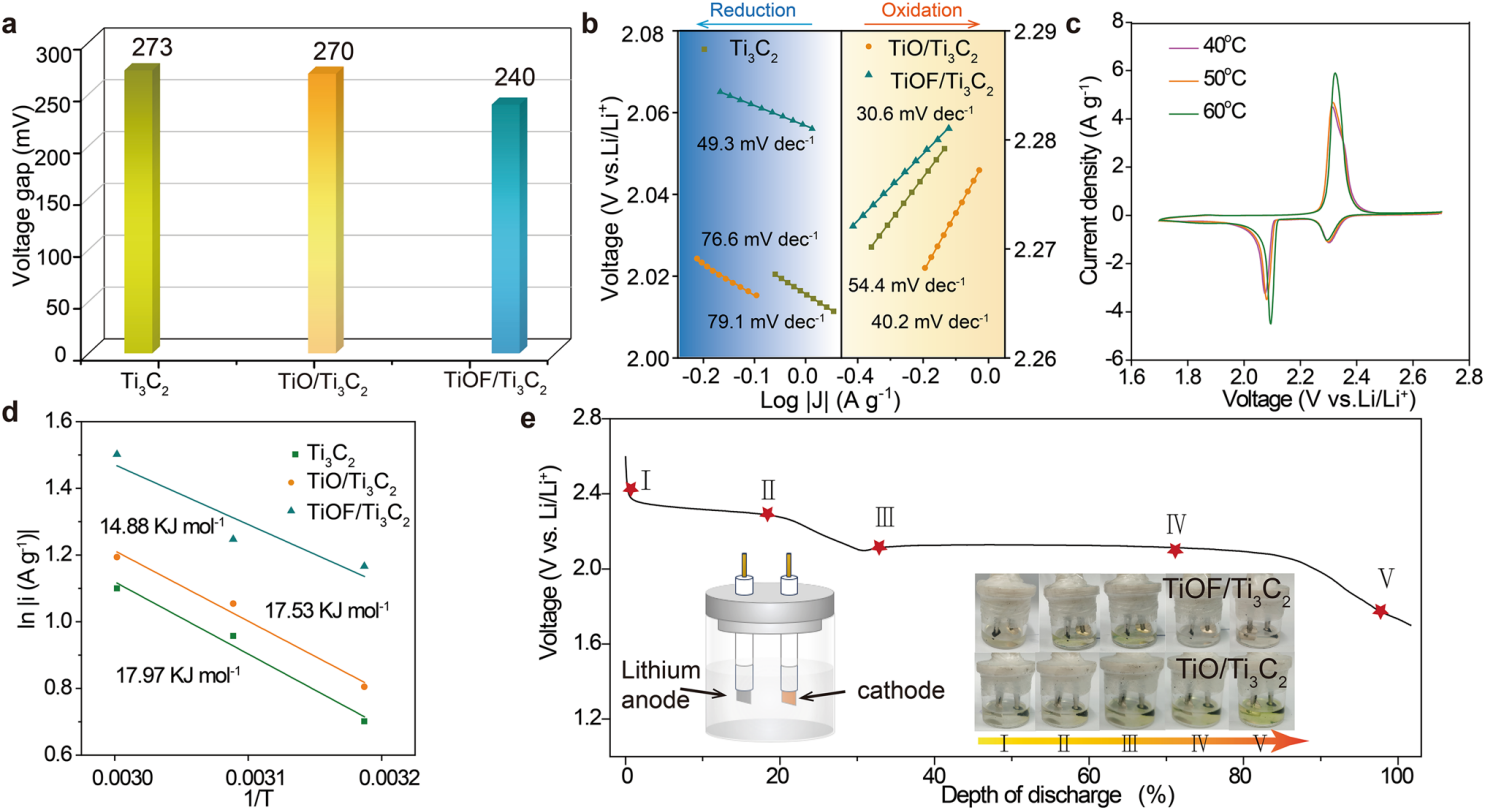
**a** Numerical statistics of the voltage gap derived from CV curves. **b** Tafel plots, **c** CV curves of Li–S batteries at 0.1 mV s^−1^ with TiOF/Ti_3_C_2_ at 40, 50, and 60 °C. **d** The relationship between the peak current of the Li_2_S_4_ conversion step in the CV curves and temperature of the batteries with TiOF/Ti_3_C_2_, TiO/Ti_3_C_2_ and Ti_3_C_2_. **e** In situ observation of the transparent electrolyte in Li–S batteries with different electrocatalysts

To elucidate the key role of catalysts, CV measurements were conducted show two reduction peaks (R1 and R2) and two oxidation peaks (O1 and O2) in the curves (Fig. S10). Analyzing the voltage gap between the O1 peak and R2 peak, it is revealed that the TiOF/Ti_3_C_2_ catalysts minimize polarization, thus facilitating the sulfur redox reaction process (Fig. 3a and Table S2). Tafel plots derived from these measurements shed light on the charge transfer kinetics. Figure 3b demonstrates that TiOF/Ti_3_C_2_ exhibits lower Tafel slopes compared to TiO/Ti_3_C_2_ and Ti_3_C_2_ in both reduction and oxidation processes, indicating the accelerated LiPSs conversion kinetic rates. To gain a deeper understanding of the role of catalysts in expediting the LiPSs conversion process, we evaluated the activation energy (E_a_), a crucial indicator of catalytic activity. Temperature-dependent CV tests conducted at varying temperatures (40, 50, and 60 °C) provided insights into the E_a_ associated with the liquid–solid reaction phase, focusing on its capacity contribution (Figs. 3c and S11). The peak current (*j*) is proportional to the reaction rate (*k*), which implies that *j* can be fitted to the Arrhenius equation *j* ∝ *k* = *A* × *e*^−Ea/RT^. According to the fits shown in Fig. 3d, the TiOF/Ti_3_C_2_ (14.88 kJ mol^−1^) has a lower E_a_ than TiO/Ti_3_C_2_ (17.53 kJ mol^−1^) and Ti_3_C_2_ (17.97 kJ mol^−1^), indicating the catalytic activity of TiOF/Ti_3_C_2_.

To intuitively visualize the catalytic capability of catalysts, in situ visual vial-cell experiments with different cathodes were constructed, and the color evolution of electrolytes was monitored upon the discharge process, as shown in Fig. 3e. The electrolyte in the TiOF/Ti_3_C_2_ setup turned a pronounced yellow at the second potential plateau (2.1 V) and faded slightly due to the precipitation of insoluble Li_2_S, eventually becoming almost colorless, indicative of efficient LiPSs conversion (Fig. 3e). In contrast, the cell with TiO/Ti_3_C_2_ exhibits no color change at the end of the discharge process. These observations corroborate that TiOF/Ti_3_C_2_ can accelerate the conversion of sulfur species, which is consistent with the kinetic behavior analysis of electrochemical reactions.

**Fig. 4:**
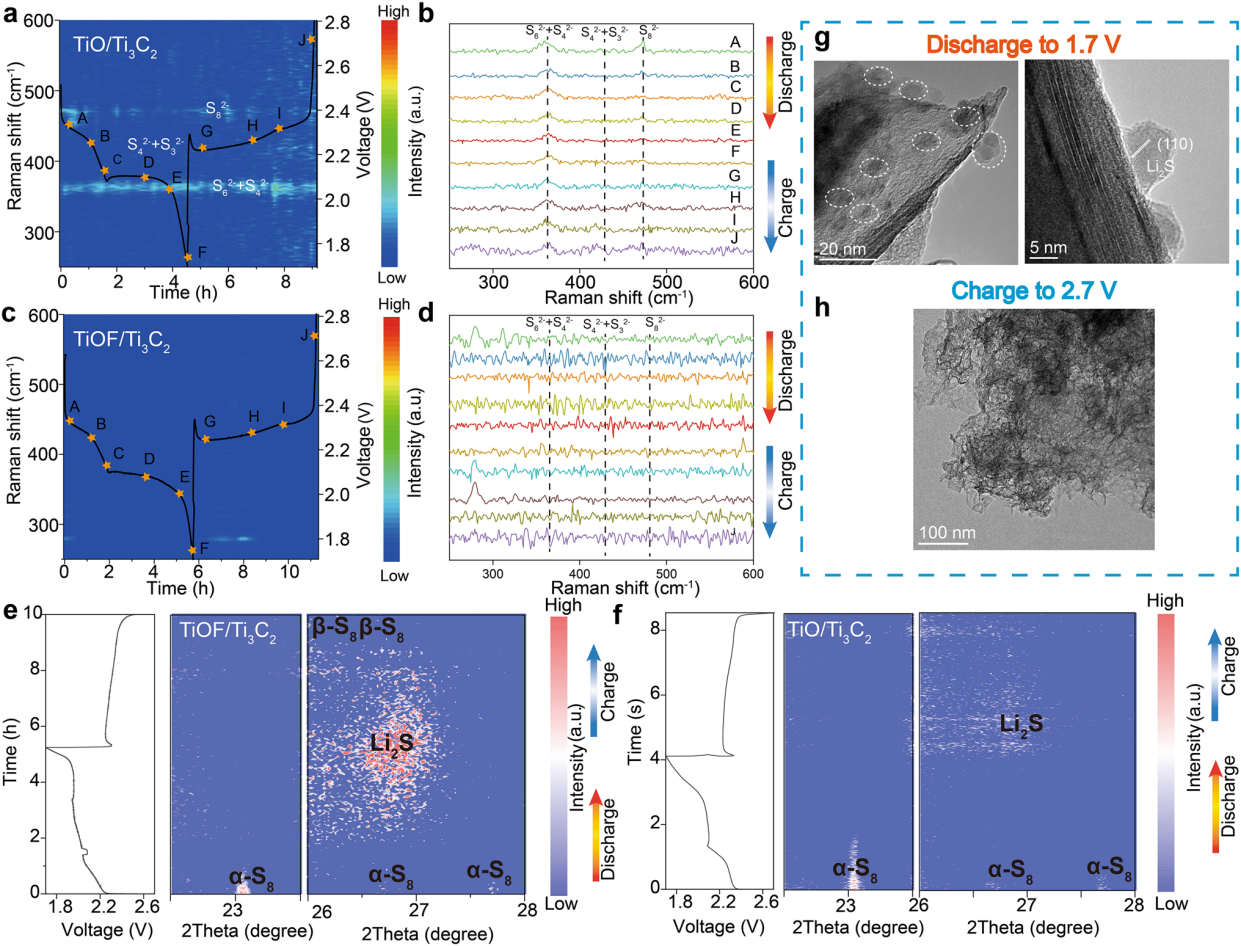
**a, c** Operando Raman images of TiO/Ti_3_C_2_ and TiOF/Ti_3_C_2_ with the first discharge–charge curves, respectively. **b, d** The selective Raman spectra of TiO/Ti_3_C_2_ and TiOF/Ti_3_C_2_. **e, f** First discharge–charge curves of the TiOF/Ti_3_C_2_ and TiO/Ti_3_C_2_ electrodes with the corresponding operando XRD contour plots. **g** TEM image and HRTEM images of TiOF/Ti_3_C_2_ at the end of the discharging. **h** TEM image of TiOF/Ti_3_C_2_ after the fully charging

Considering the dynamic and process-dependent nature of the catalytic mechanisms in Li–S batteries, coupled with enclosed reaction processes, we have employed the in situ method to evaluate the effects of inhibiting the shuttle phenomenon using different electrode materials. As shown in Fig. S12, the device construction diagram illustrates real-time qualitative detection of LiPSs through a quartz observation window. To enable laser illumination into the cathode and capture Raman signals from LiPSs, a hole was introduced in the negative case. Figure 4 presents operando time-resolved Raman images of LiPSs in Li–S batteries with TiOF/Ti_3_C_2_ and TiO/Ti_3_C_2_ electrodes at different voltages states. During the discharge process, with the TiO/Ti_3_C_2_ electrode (Fig. 4a, b), signals corresponding to S_8_^2−^ (peaks located at 478 cm^−1^) were detected at the initial stages, indicating the formation of long-chain LiPSs [58]. Additionally, peaks around 364 cm^−1^ represented LiPSs such as Li_2_S_4_ + Li_2_S_6_. Throughout the charging process, the characteristic S_4_^2−^ + S_6_^2−^ and S_3_^2−^ peaks remained, suggesting shuttle effect and irreversible loss of LiPSs [59]. Conversely, almost no LiPSs signals were observed during both discharge and charge processes with the TiOF/Ti_3_C_2_ electrode (Fig. 4c, d), indicating the effective prevention of the shuttling.

Different from in situ Raman spectroscopy, which focuses on the evolution of intermediates during charge and discharge processes, in situ XRD spectroscopy is used to monitor the information of sulfur species, such as crystal structure, phase transitions and reaction kinetics [60]. Figure S13 elucidates the device construction diagram, illustrating a beryllium observation window was anchored for real-time detection of LiPSs. The intensity of the initial α-S_8_ phases gradually diminishes as the discharge progresses, accompanied by a noticeable increase in the Li_2_S peak at approximately 27° (Fig. 4e, f). It is noteworthy that the nucleation of Li_2_S appears in the early stage of the second discharge platform and fully transforms into the β-S_8_ phase, as evidenced by the prominent strong peaks observed after charging. In comparison, even at the end of the discharge process, only faint Li_2_S peaks are detected. These distinct differences highlight that TiOF/Ti_3_C_2_ with abundant active sites can facilitate the rapid and complete transition between sulfur and Li_2_S. Combining the results of TEM characterizations, TiOF/Ti_3_C_2_ forms numerous crystalline Li_2_S particles at the end of the discharge process, which is consistent with the in situ XRD results. After full charging, TiOF/Ti_3_C_2_ still maintains its three-dimensional layered structure, demonstrating the stability of structure. Therefore, it not only effectively addresses the well-known issue of LiPSs shuttling but also enhances the utilization efficiency of sulfur.

### Analysis of Catalytic Activity Origin in Redox Reactions

**Fig. 5:**
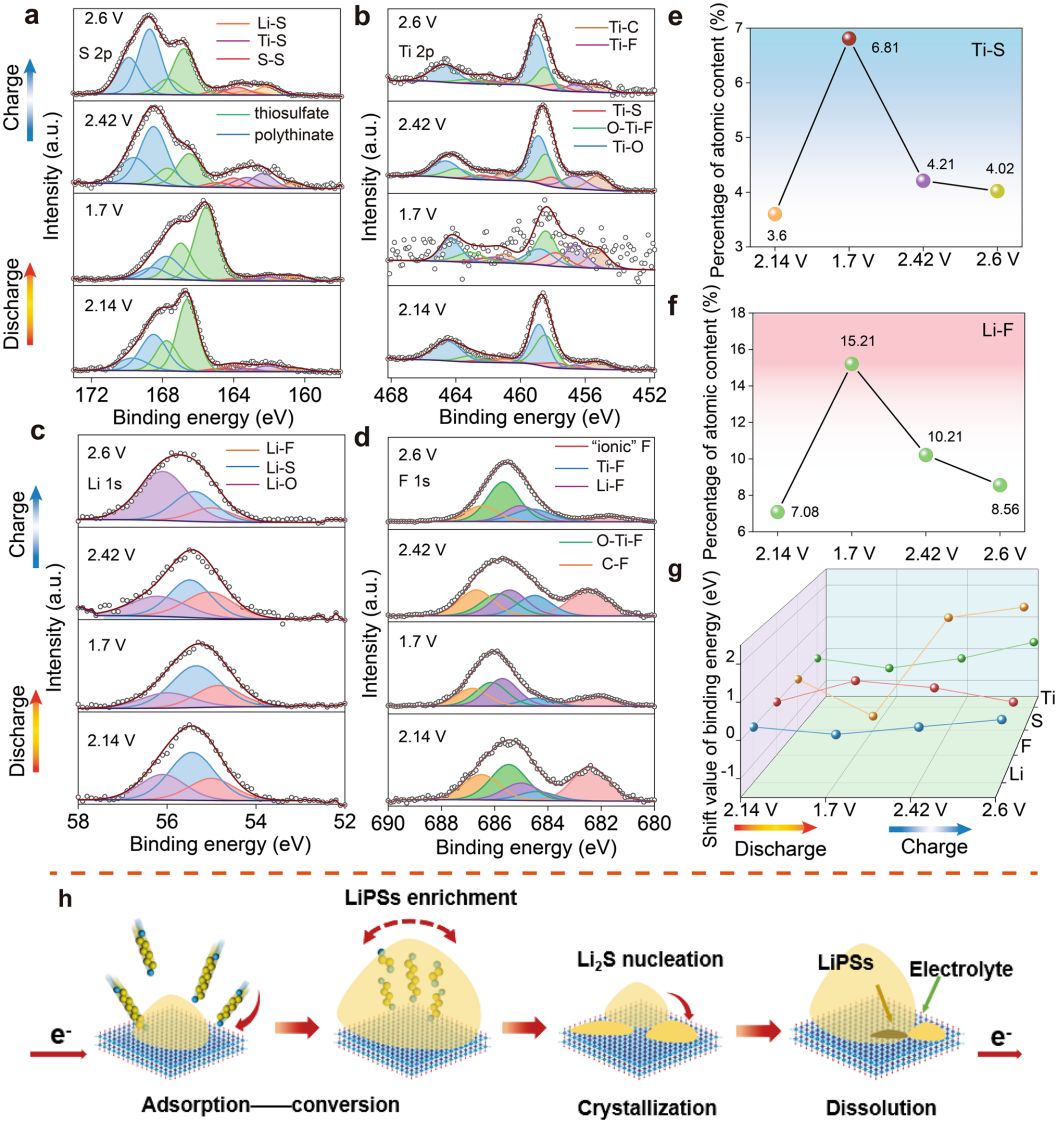
Ex situ XPS spectra analysis. XPS spectra of **a** S 2*p*, **b** Ti 2*p*, **c** Li 1*s* and **d** F 1*s* on the TiOF/Ti_3_C_2_ electrodes at different discharge/charge states. Atomic percentage analysis of **e** Ti-S and **f** Li–F. **g** Shift values of binding energy for Li, F, S and Ti at different discharge/charge states. **h** Catalytic mechanism of the LiPSs on the surface of TiOF/Ti_3_C_2_ during the multiple consecutive heterogeneous catalytic processes

To elucidate the catalytic mechanism of TiOF/Ti_3_C_2_ catalysts, we performed ex situ XPS measurements at the selected states of the charge/discharge processes to investigate the role of fluorine modulation in enhancing the sulfur reduction and Li_2_S oxidation processes (Figs. 5 and S14). In Fig. 5a, several sub-bands corresponding to Li–S, S–S, Ti–S, thiosulfates, and polythionate were observed, indicating that the inherent chemical interaction between Ti and S. This interaction is further substantiated by the high-resolution XPS of Ti 2*p* (Fig. 5b). It displays five characteristic peaks at 455.2, 456.6, 457.8, 458.4, and 458.8 eV, corresponding to Ti–C, Ti–F, Ti–S, O–Ti–F, and Ti–O bonds, respectively [45, 47]. Notably, during the discharge process, the binding energy of Ti 2*p* shifts to lower values as sulfur species undergo reduction, highlighting the cleavage of S–S bonds and the formation of Li–S bonds during the discharge process. The three-dimensional layered structure of TiOF/Ti_3_C_2_ effectively exposes Ti metal sites, and modulation by highly electronegative fluorine elements enhances the positive charge on Ti, facilitating the formation of Ti–S bonds with LiPSs. To further elucidate the reaction mechanism, the theoretical calculations were conducted. The electron localization function (ELF) of TiOF/Ti_3_C_2_, TiO/Ti_3_C_2_, and Ti_3_C_2_ is shown in Fig. S15. As for the TiOF/Ti_3_C_2_ catalyst, the areas surrounding the Ti atoms exhibit higher ELF values, indicating a greater degree of electron localization in space, which suggests that the bonding nature between metal atoms is similar to covalent bonds. According to Bader charge analysis, due to the electron-withdrawing effect of F, Ti atoms in the TiOF/Ti_3_C_2_ catalyst lose 1.9409 electrons, resulting in a more electron-deficient state compared to TiO/Ti_3_C_2_ and Ti_3_C_2_ (Table S3). The electron-deficient state facilitates stronger interactions with polysulfides, thereby enhancing the catalytic activity [61, 62]. This promotes the disruption of S–S bonds within sulfur species, reducing the nucleation barrier for Li_2_S and decreasing the activation energy for the reaction. The formation of Ti–S bonds also involves a portion of Li^+^ migrating to the sites occupied by F, resulting in the creation of a stable inorganic LiF interfacial layer on the surface of the sulfur cathode [35, 63, 64]. In conjunction with the preceding electrochemical, spectroscopic, and imaging analyses, these findings imply that this interfacial layer plays a role in promoting uniform nucleation and the generation of Li_2_S particles. It also strengthens the interaction between the host material and Li_2_S, thereby optimizing the transport of electrons and ions. As shown in Fig. 5c, d, several sub-bands corresponding to Li–F, Li–S and Li–O of Li 1*s* were observed. Simultaneously, the F 1*s* spectrum can be divided into “ionic” F, Ti–F, Li–F, O–Ti–F, and C–F [31, 65]. The peak of “ionic” F represents fluorine ions from the electrolyte that adhere to the electrode surface. The charging process involves the breaking of Li–S bonds and the formation of S–S bonds, accompanied by the release of Li^+^ and the overflow of electrons. The uniformly dispersed F atoms, possessing high electronegativity, effectively capture escaping electrons through space charge compensation. Compared with F 1*s* of TiO/Ti_3_C_2_, the shift of binding energy is not obvious (Fig. S16). As shown in Fig. 5e–g, through conducting a semi-quantitative analysis of XPS data, we have observed variations in the atomic percentage content and peak positions of Ti-S bonds and Li–F bonds (Table S4). These observations suggest that the electron transfer process is reversible, indirectly implying the stability and sustainability of the catalytic process during the multiple consecutive heterogeneous catalytic processes (Fig. 5h).

### Electrochemical Performance of Li–S Batteries with the TiOF/Ti_3_C_2_ Catalyst

**Fig. 6:**
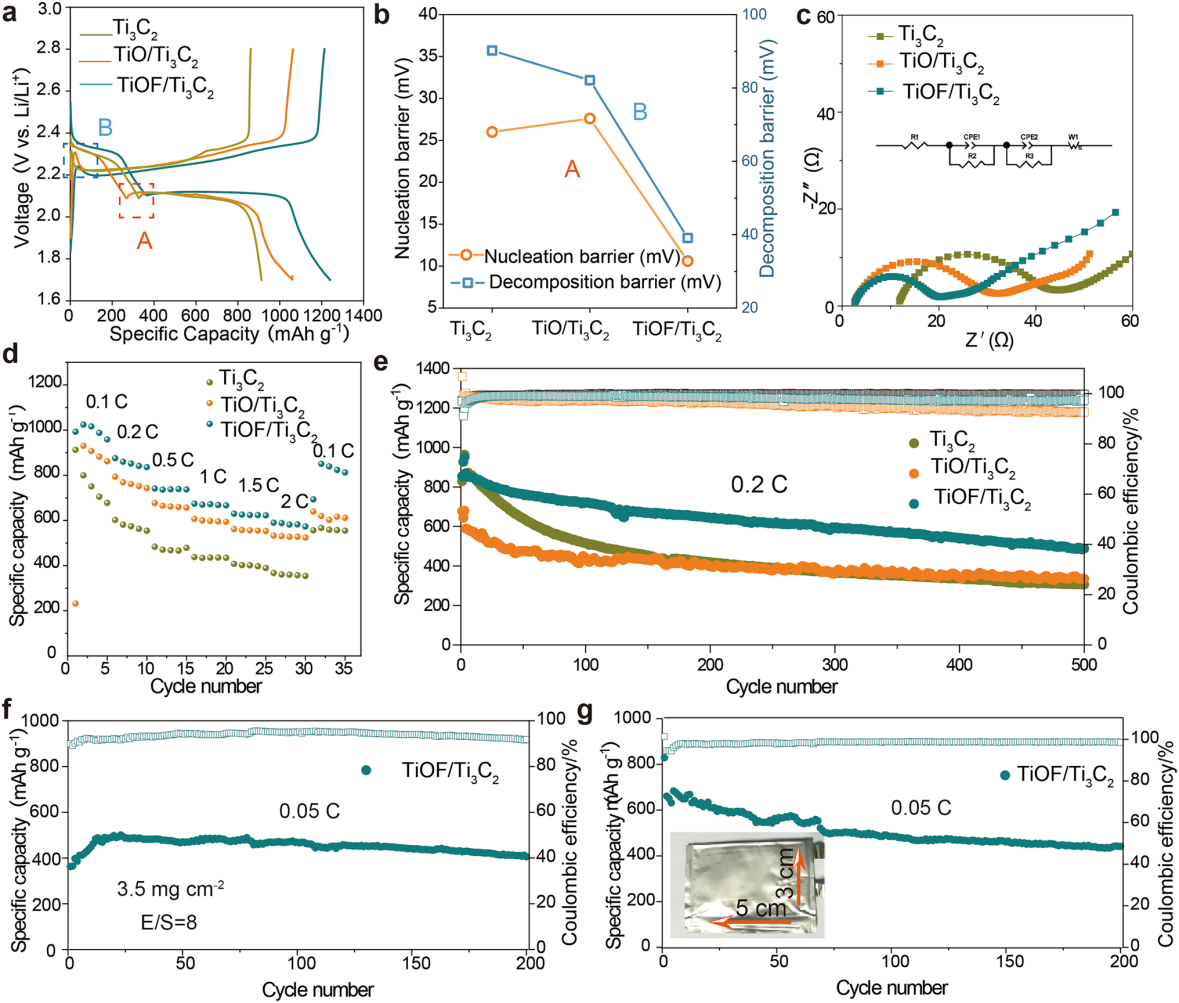
Optimized electrochemical performance with TiOF/Ti_3_C_2_ catalysts.** a** Galvanostatic charge/discharge profiles at 0.2 C. **b** Nucleation overpotentials at the onset of 2.1 V plateaus and decomposition overpotentials at the beginning of the charging process. **c** Nyquist curves for Li–S batteries with different catalysts. **d** Rate performance of Li–S batteries with different catalysts.** e** Cycling performance of Li–S batteries with different catalysts at 0.2 C for 400 cycles. **f** Cycling performance with a high sulfur mass loading of 3.5 mg cm^−2^ at 0.05 C. **g** Cycling performance of the large pouch cell with the TiOF/Ti_3_C_2_ cathode

To insight into the positive impact of the modulating fluorine element of Ti_3_C_2_T_x_ MXenes catalyst, the electrochemical properties of the Li–S batteries are shown in Fig. 6. As displayed in Fig. 6a, TiOF/Ti_3_C_2_ achieved the highest capacity of 1240 mAh g^−1^ at 0.1 C among all the properties of Li–S batteries. Figure 6b summarizes nucleation overpotentials at the initiation of 2.1 V plateaus and decomposition overpotentials at the beginning of the charging process. These overpotentials are often considered as kinetic indicators in the redox reaction process since they represent the energy barriers that must be overcome during the solid–liquid phase transition of sulfur species. Notably, TiOF/Ti_3_C_2_ exhibits the lowest barrier in both nucleation and decomposition processes. The optimization of electron transport in the cathode was also evaluated by electrochemical impedance spectra (EIS) with an amplitude signal of 5 mV over a frequency range of 200 kHz-10 mHz. Figure 6c shows that the smallest diameter for TiOF/Ti_3_C_2_, indicating its superior catalytic activity. Figure 6d illustrates the electrochemical performance of Li–S batteries with various cathodes at different current densities. As expected, the TiOF/Ti_3_C_2_ cathode demonstrates excellent rate capacities, surpassing those of TiO/Ti_3_C_2_ and Ti_3_C_2_ cathodes at each current density. Under high current density, the TiOF/Ti_3_C_2_ cathode exhibits a specific capacity of 384 mAh g^−1^ at 3 C and 304 mAh g^−1^ at 5 C (Fig. S17). For the long-term cycling performance, TiOF/Ti_3_C_2_ batteries with sulfur loading of 1.5 mg cm^−2^ deliver the initial and retained discharge capacities of 868 and 486 mAh g^−1^ after 500 cycles, corresponding to a capacity decay ratio of 0.088% per cycle. In contrast, TiO/Ti_3_C_2_ and Ti_3_C_2_ batteries exhibit a lower capacity retention ratio (Fig. 6e). Furthermore, separators and the lithium anodes of batteries after cycling were also observed (Figs. S18 and S19). Considering that Li is sensitive to oxygen and humidity from the air, an airtight transfer box was used to protect the Li anodes after cycling in an argon-filled glove box, ensuring that the excellent performance of TiOF/Ti_3_C_2_ is due to the inhibition of the shuttle effect. Additionally, the particles observed on the surface of the lithium deposition morphology are likely due to the non-uniformity of lithium deposition. The uneven current density distribution during the deposition process can cause lithium to form a granular structure.

When the sulfur mass loading was increased to 3.5 mg cm^−2^ with an electrolyte/sulfur (E/S) ratio of 8:1, the TiOF/Ti_3_C_2_ battery still exhibited a high capacity retention of 74.1% after 200 cycles (Fig. 6f). When the sulfur mass loading was increased to 6.71 mg cm^−2^ with an electrolyte/sulfur (E/S) ratio of 8:1, the TiOF/Ti_3_C_2_ battery exhibited capacity retention of 86.5% after 60 cycles (Fig. S20). The radar chart shows that the performance comparison with the reported works (Fig. S21) [49, 66–71]. On this basis, a pouch cell with sulfur loading of 1.5 mg cm^−2^ and an E/S ratio of 21  was assembled to approach the realistic conditions required for practical Li–S batteries. As shown in Fig. 6g, TiOF/Ti_3_C_2_ batteries delivered initial and retained discharge capacities of 683 and 441 mAh g^−1^, maintaining a capacity retention of 64.5% after 200 cycles. Hence, the electrochemical results demonstrate the superior catalytic activity of the TiOF/Ti_3_C_2_ catalysts in practical Li–S batteries applications.

## Discussion

Utilizing fluorination to address the shuttle effect and the sluggish redox reaction kinetics, TiOF/Ti_3_C_2_ MXene nanoribbons, as fluorinated MXene derivatives, were prepared by NH_4_F pyrolysis treatment, which presented in creating a highly active and directional pathway for Li–S redox reaction via catalysis. F^−^ induced O–Ti–F covalent bonds increase the positive charge on Ti metal sites due to the Lewis acid-based mechanism, which lead to a stronger interaction between the active site of Ti and polysulfides. As a result, TiOF/Ti_3_C_2_ MXene facilitates the deposition of Li_2_S at lower overpotentials. Simultaneously, the formation of a LiF interface layer contributes to the electrode stability, provides higher electronic/ionic conductivity and ultimately expedites the liquid–solid phase transition during the discharge process. During the breaking of Li–S bonds in the charging process, uniformly distributed F atoms capture the electrons by charge compensation mechanisms, which can accelerate the escape of Li^+^ and further obtain a lower activation energy for solid–liquid phase transitions. From a methodological perspective, the results will provide an understanding of the efficient coupling of multiphase sulfur species conversion. This renewed mechanistic insight reveals an underlying principle that may serve as a reference model for a wider range of fluoride-regulated catalysts.

## Supplementary Information

Below is the link to the electronic supplementary material.

Supplementary file1 (DOCX 5120 KB)
