# Supplementary material for: Fluorine-Modulated MXene-Derived Catalysts for Multiphase Sulfur Conversion in Lithium–Sulfur Battery
Source: Nanomicro Lett. 2024 Aug 12;16:266. doi: 10.1007/s40820-024-01482-6 (PMC11319705; doi:10.1007/s40820-024-01482-6)
Supplement: Supplementary file 1 — Supplementary file1 (DOCX 5120 KB) [file 40820_2024_1482_MOESM1_ESM.docx]

Supporting Information for

**Fluorine Modulated MXene-derived Catalysts for Multi-Phase Sulfur Conversion in Lithium-Sulfur Battery**

Qinhua Gu^1,2^, Yiqi Cao^1,3^, Junnan Chen^1,2^, Yujie Qi^1^, Zhaofeng Zhai^1^, Ming Lu^1,3,^*, Nan Huang^1,2^, Bingsen Zhang^1,2,^*

^1^ Shenyang National Laboratory for Materials Science, Institute of Metal Research, Chinese Academy of Sciences, Shenyang 110016, P. R. China

^2^ School of Materials Science and Engineering, University of Science and Technology of China, Shenyang 110016, P. R. China

^3^ The Joint Laboratory of MXene Materials, Key Laboratory of Functional Materials Physics and Chemistry of the Ministry of Education, Key Laboratory of Preparation and Application of Environmental Friendly Materials of the Ministry of Education, Jilin Normal University, Changchun 130103, P. R. China

*Corresponding authors. Email: [luming@jlnu.edu.cn](mailto:luming@jlnu.edu.cn) (Ming Lu); [bszhang@imr.ac.cn](mailto:bszhang@imr.ac.cn) (Bingsen Zhang)

**Supplementary Figures and Tables**


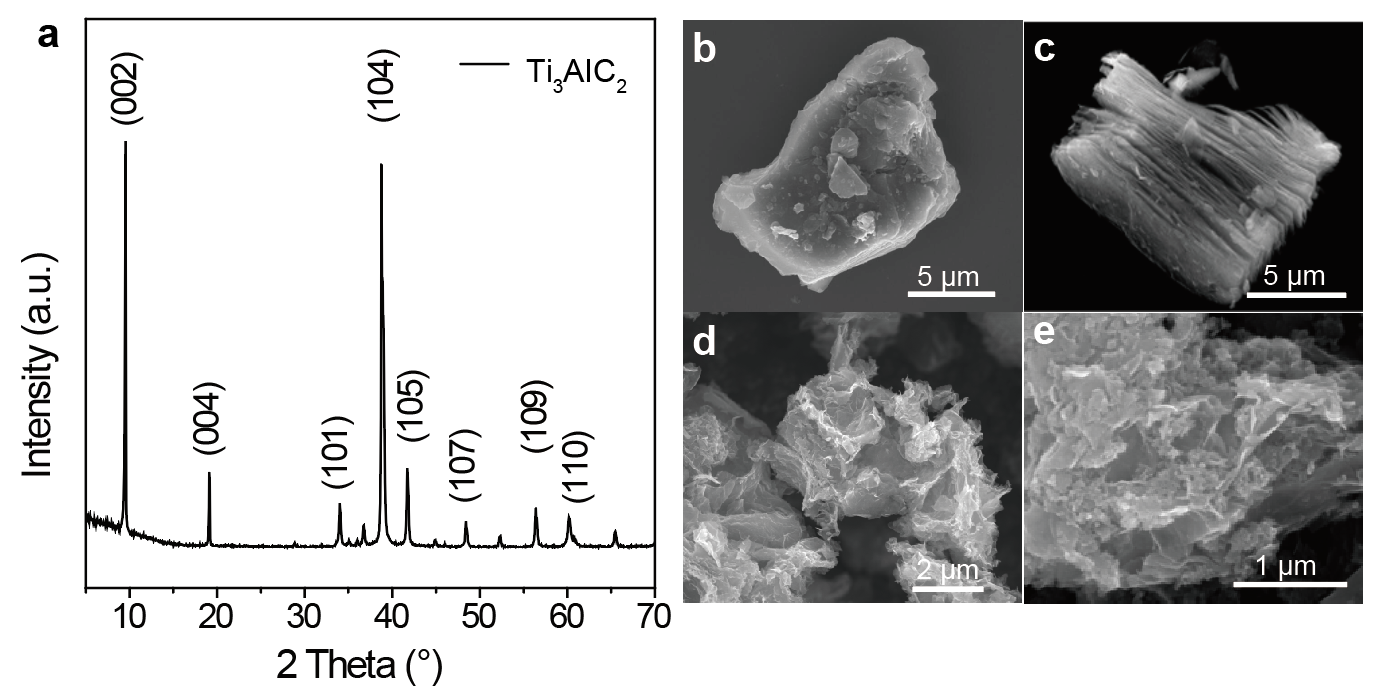


**Fig. S1** **a** XRD pattern of Ti_3_AlC_2_. SEM images of **b** Ti_3_AlC_2_ MAX, **c** multilayer Ti_3_C_2_, **d** TiO/Ti_3_C_2_ powders, and **e** TiO/Ti_3_C_2_ powders


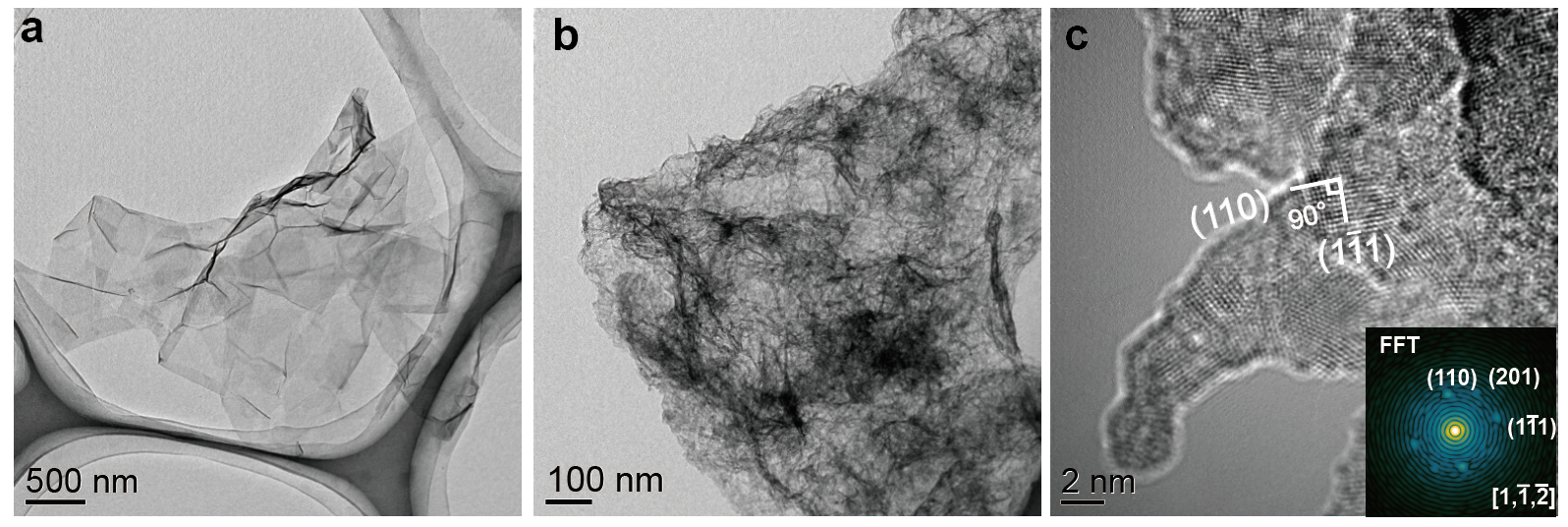


**Fig. S2** TEM images of **a** Ti_3_C_2_ nanosheets and **b** TiO/Ti_3_C_2_. **c** HRTEM images of TiO/Ti_3_C_2_ (The inset is corresponding to local FFTs)


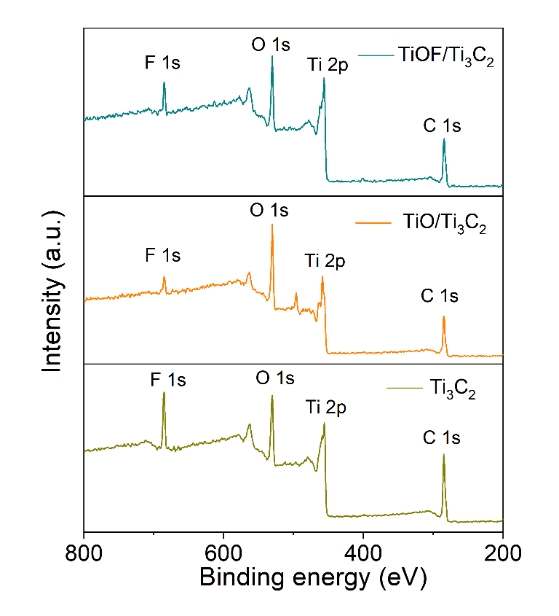


**Fig. S3** XPS spectra of Ti_3_C_2_, TiO/Ti_3_C_2_, and TiOF/Ti_3_C_2_


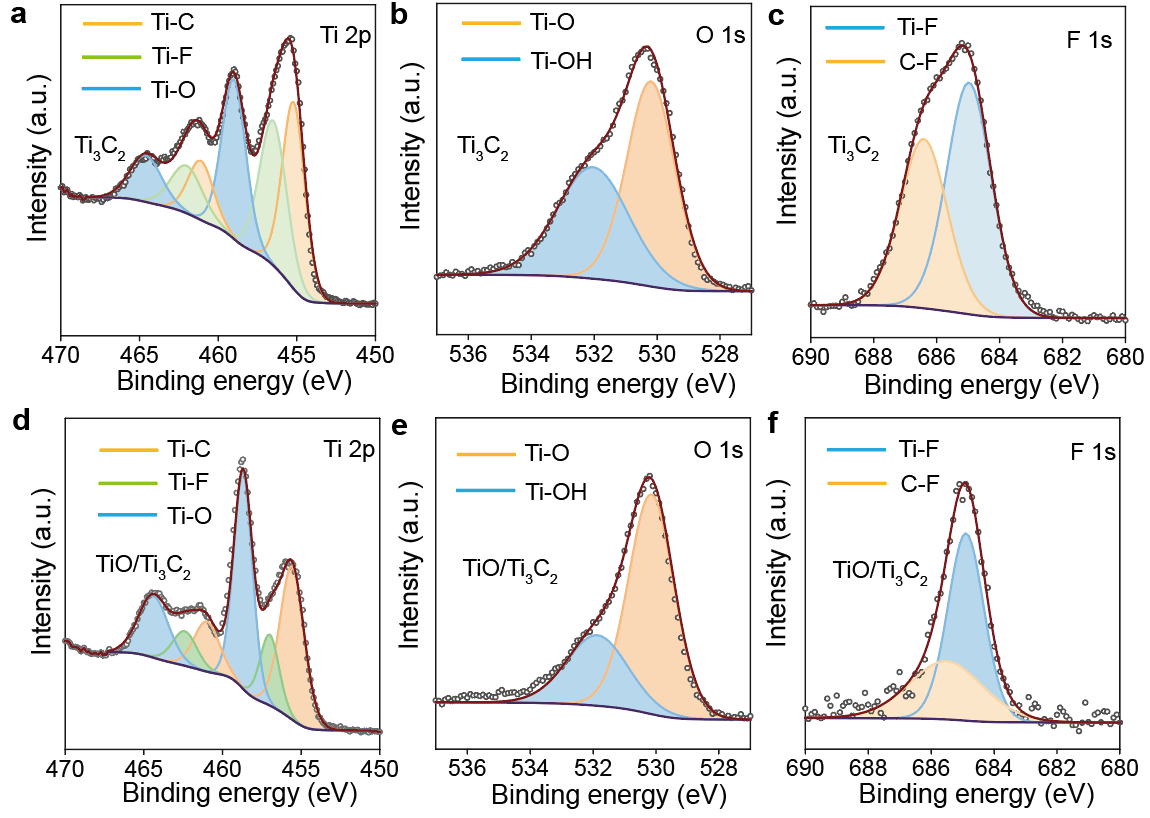


**Fig. S4** XPS spectra of **a**, **d** Ti 2p, **b**, **e** O 1s, and **c**, **f** F 1s for Ti_3_C_2_ and TiO/Ti_3_C_2_


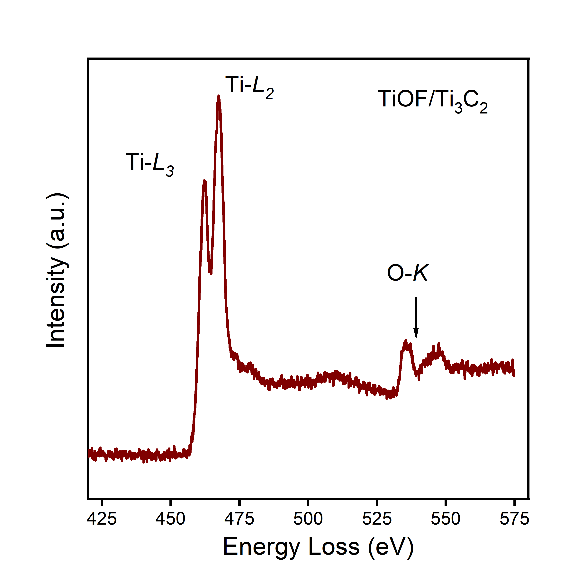


**Fig. S5** EELS spectra of TiOF/Ti_3_C_2_


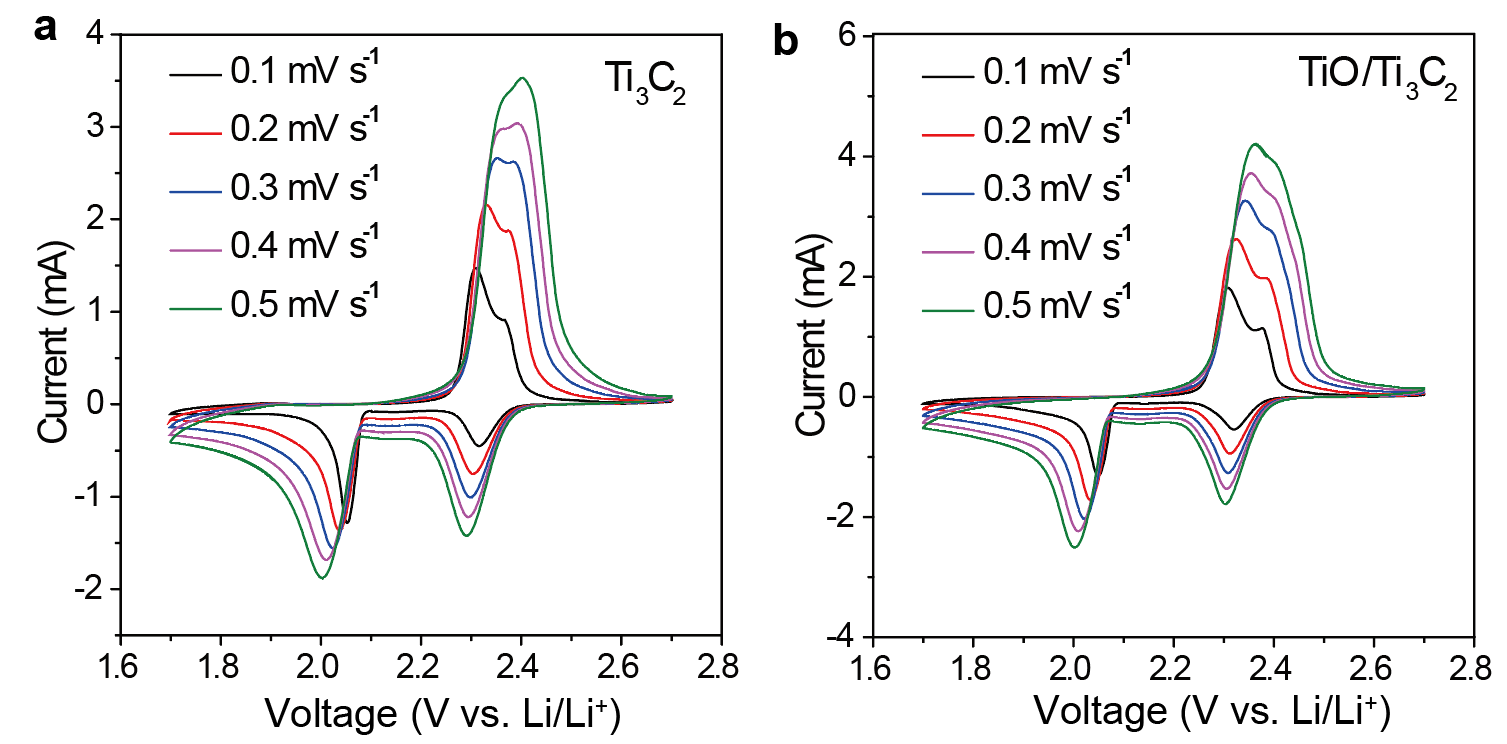
**Fig. S6** CV curves of **a** Ti_3_C_2_ and **b** TiO/Ti_3_C_2_ batteries at scan rates from 0.1 to 0.5 mV s^-1^


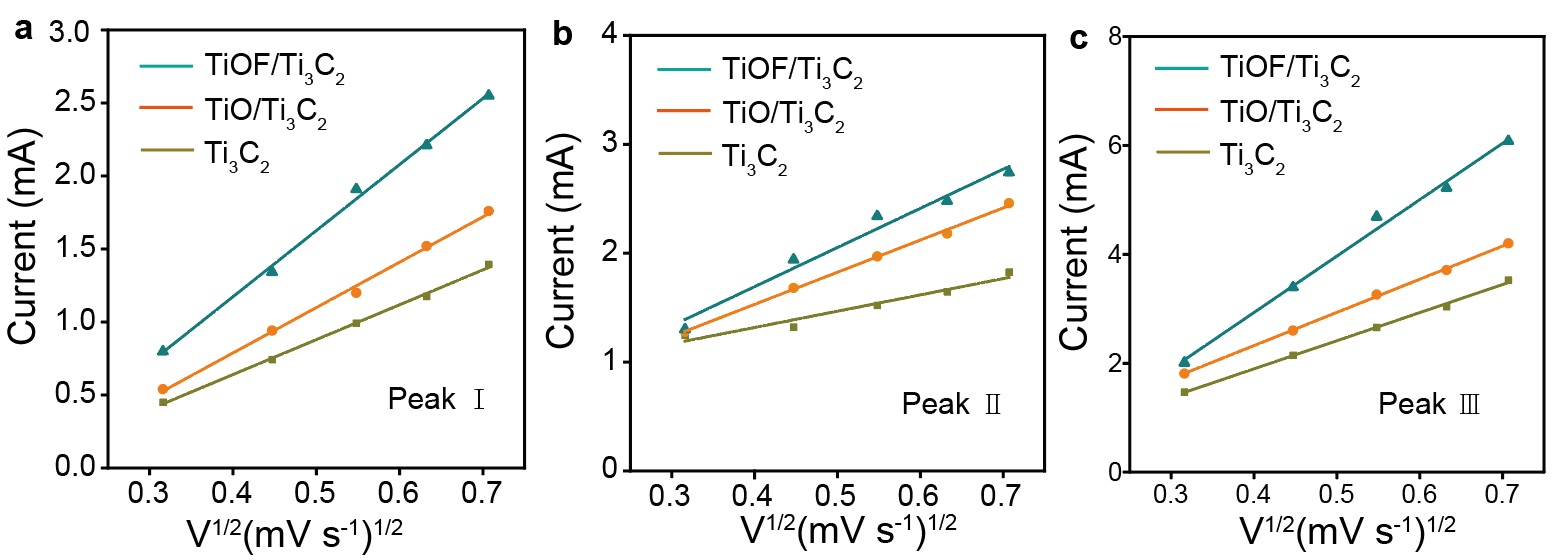
**Fig. S7** Linear fits of the peak current of **a** peak Ⅰ, **b** peak Ⅱ and **c** peak Ⅲ from CV curves


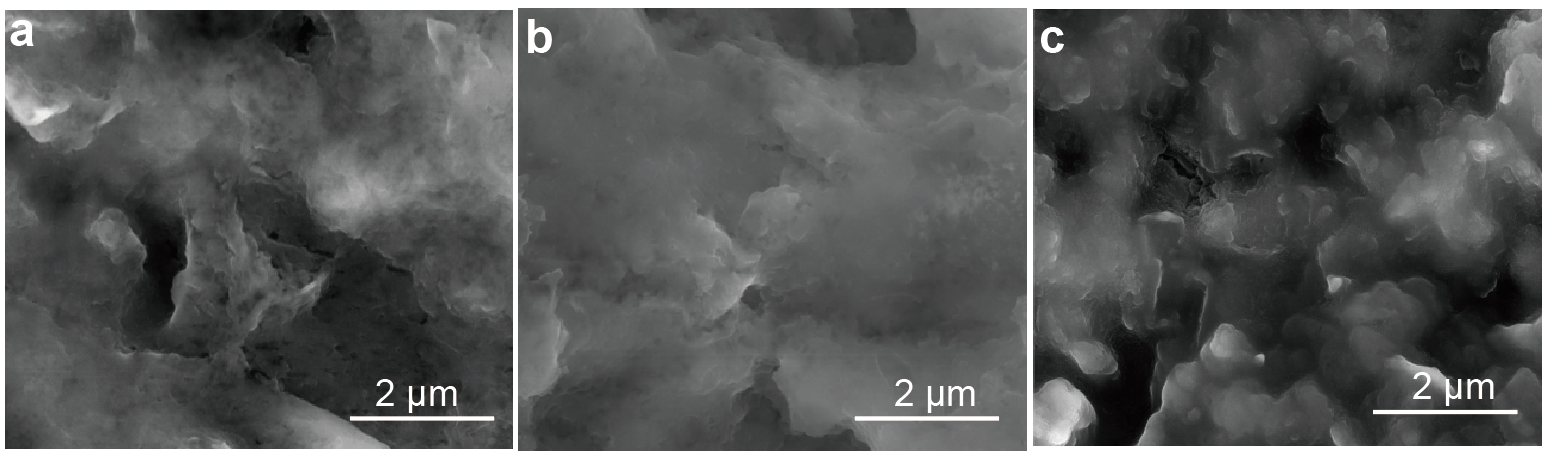


**Fig. S8** SEM images of the Li_2_S deposition morphology on **a** TiOF/Ti_3_C_2_, **b** TiO/Ti_3_C_2_, and **c** Ti_3_C_2_


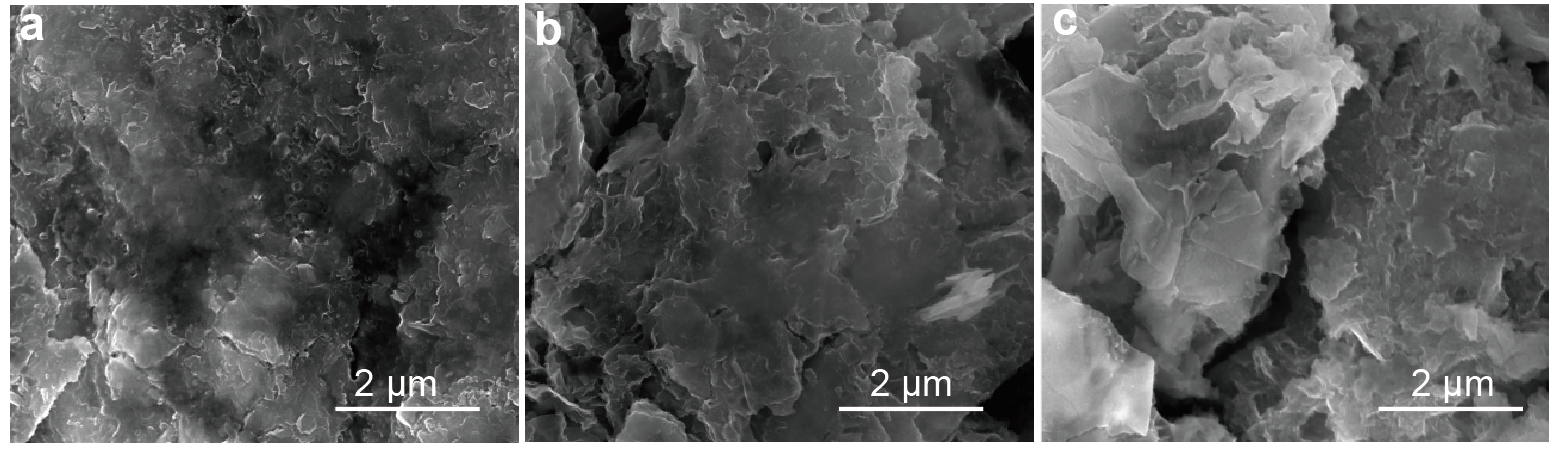


**Fig. S9** SEM images of the Li_2_S dissolution morphology on **a** TiOF/Ti_3_C_2_, **b** TiO/Ti_3_C_2_, and **c** Ti_3_C_2_


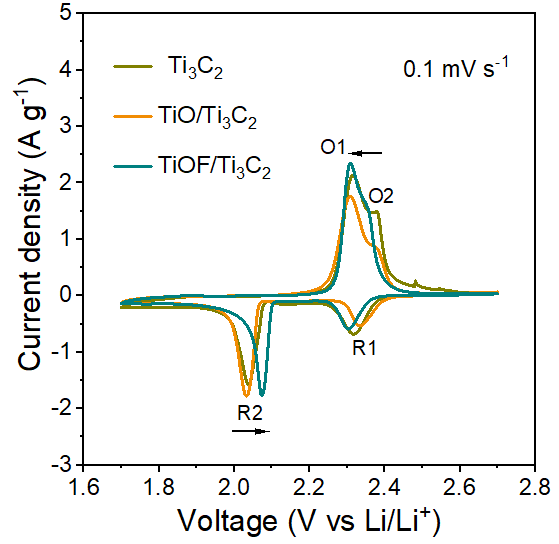


**Fig. S10** CV curves of Ti_3_C_2_, TiO/Ti_3_C_2_, and TiOF/Ti_3_C_2_ batteries


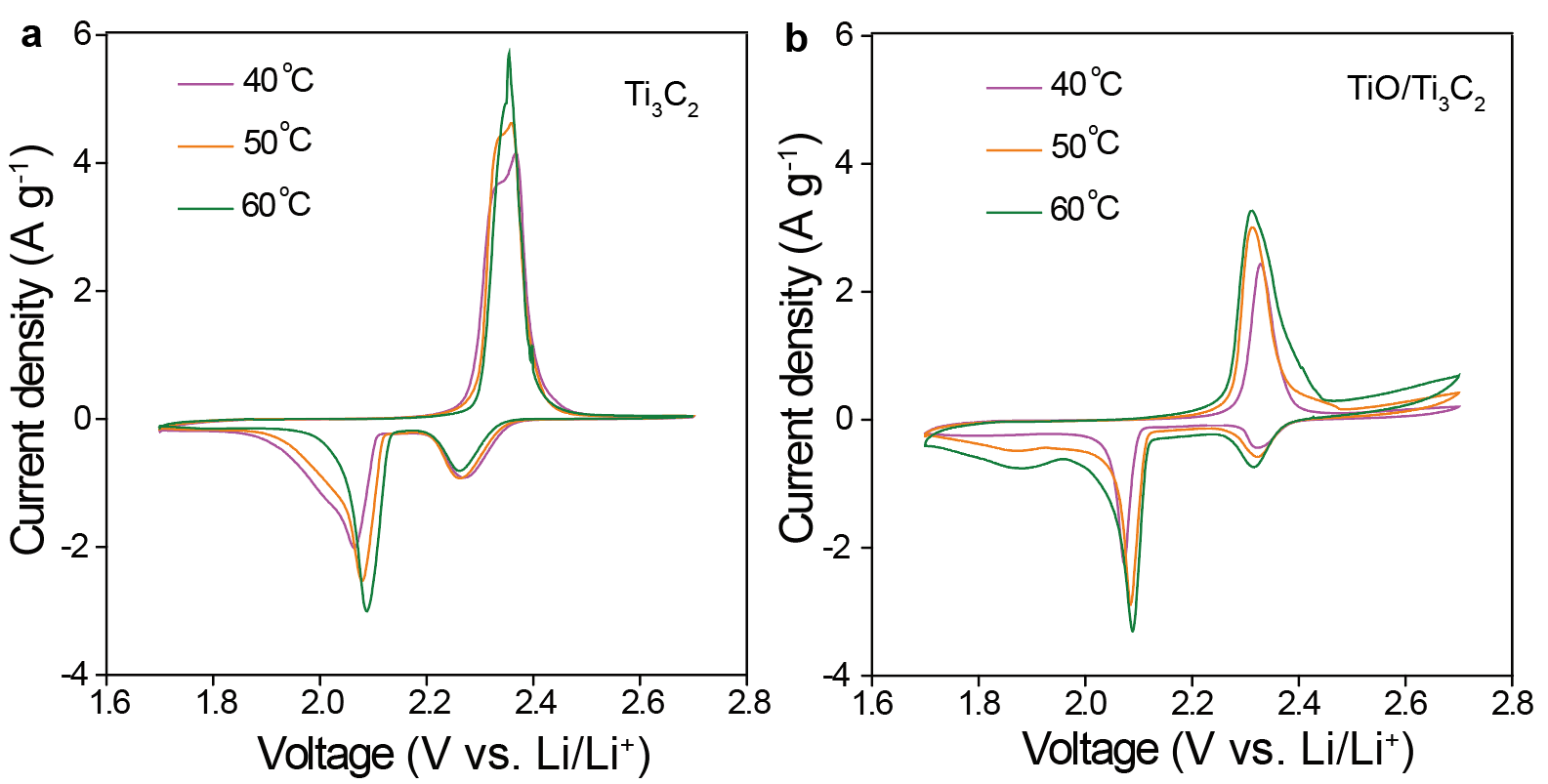


**Fig. S11** CV curves at 0.1 mV s^−1^ with **a** Ti_3_C_2_ and **b** TiO/Ti_3_C_2_ at 40, 50, and 60 °C


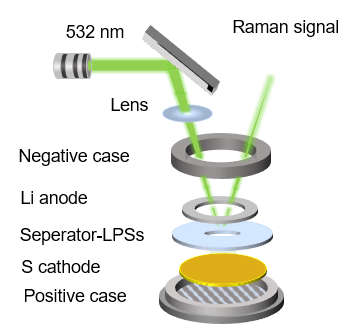


**Fig. S12** Schematic diagram of the battery assembly for operando Raman spectroscopy.


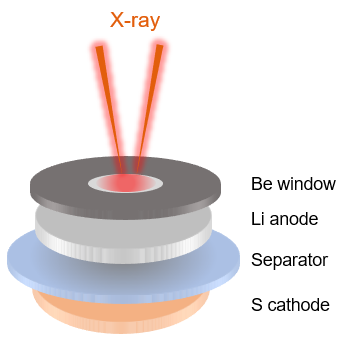


**Fig. S13** Schematic diagram of the battery assembly for operando XRD spectroscopy


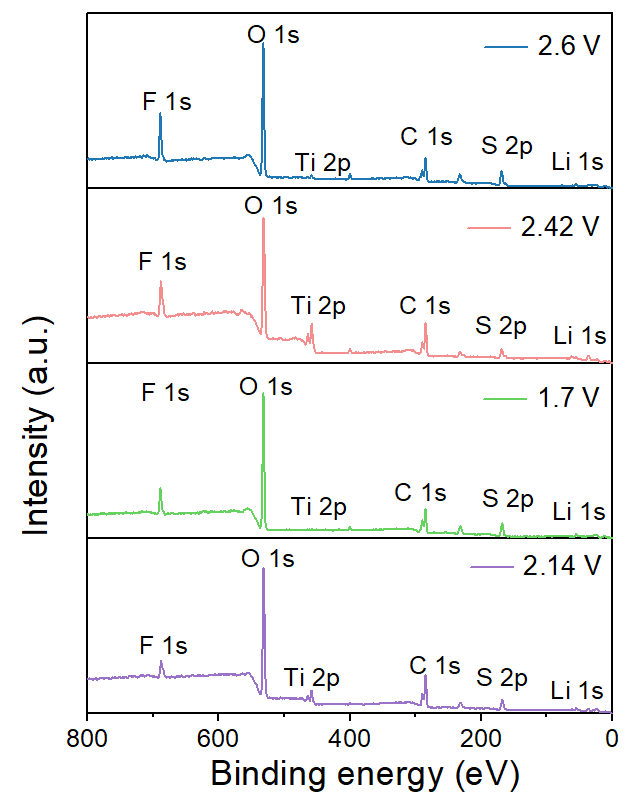


**Fig. S14** XPS of TiOF/Ti_3_C_2_-Li_2_S_6_ at different discharge/charge states


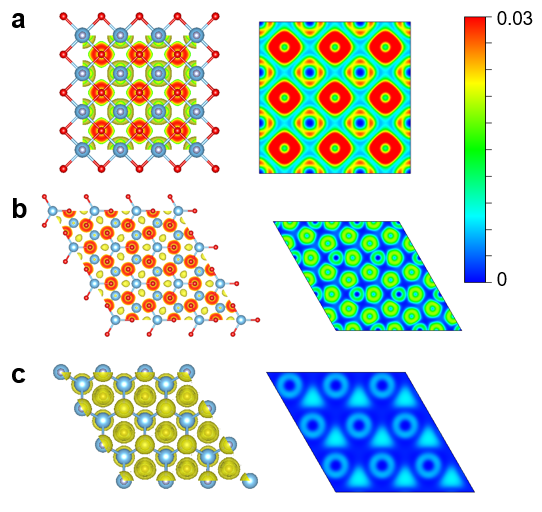


**Fig. S15** ELF of **a** TiOF/Ti_3_C_2_, **b** TiO/Ti_3_C_2_, and **c** Ti_3_C_2_


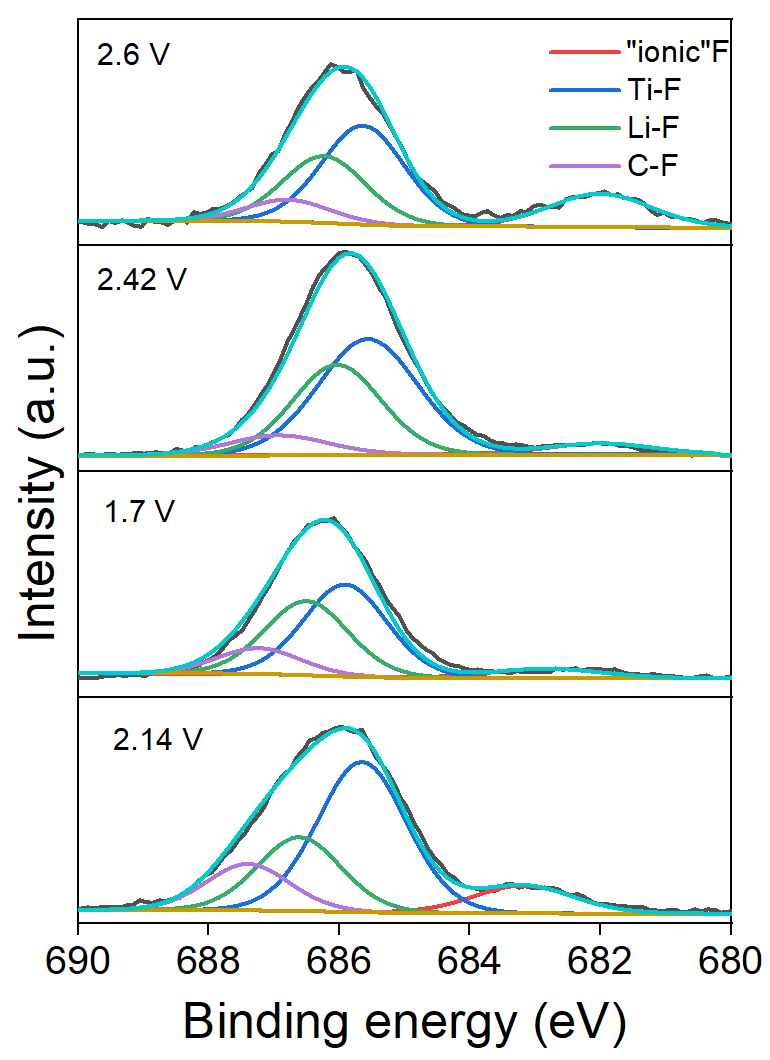


**Fig. S16** XPS of F 1s on the TiO/Ti_3_C_2_-Li_2_S_6_ at different discharge/charge states


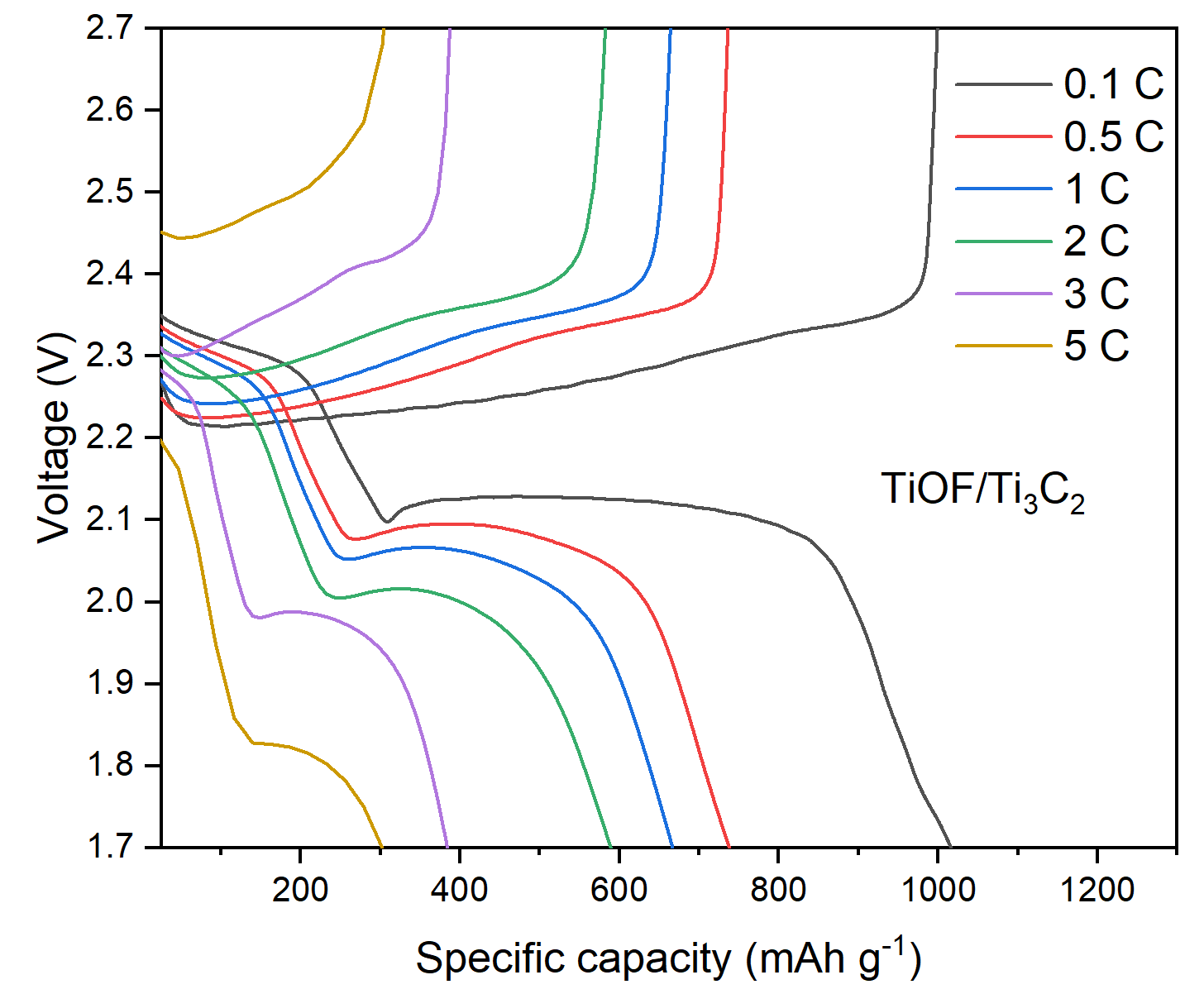


**Fig. S17** Charge/discharge curves of TiOF/Ti_3_C_2_ cathodes at different current rates


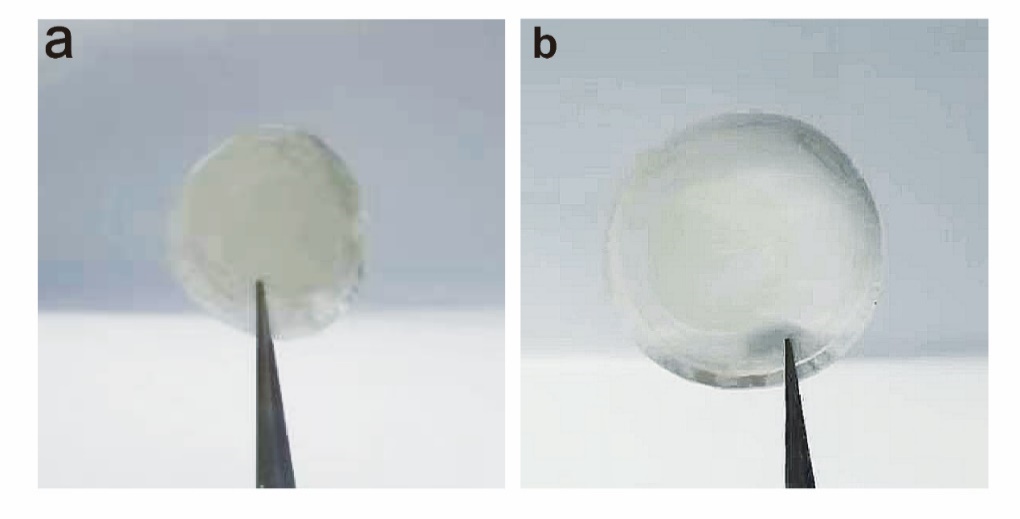


**Fig. S18** Optical photos of separators **a** with the TiO/Ti_3_C_2_ cathode and **b** with the TiOF/Ti_3_C_2_ cathode after 500 cycles at 0.2 C


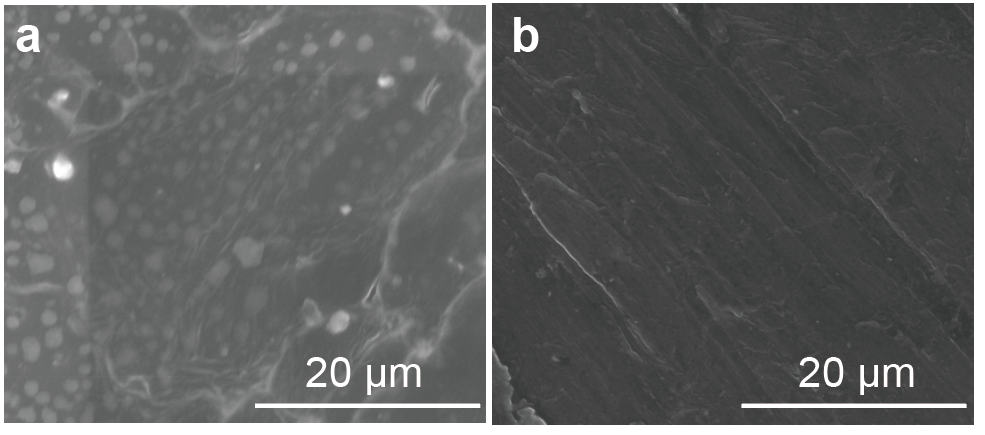


**Fig. S19** SEM images of Li anodes of batteries **a** with the TiO/Ti_3_C_2_ cathode and **b** with the TiOF/Ti_3_C_2_ cathode after 500 cycles at 0.2 C


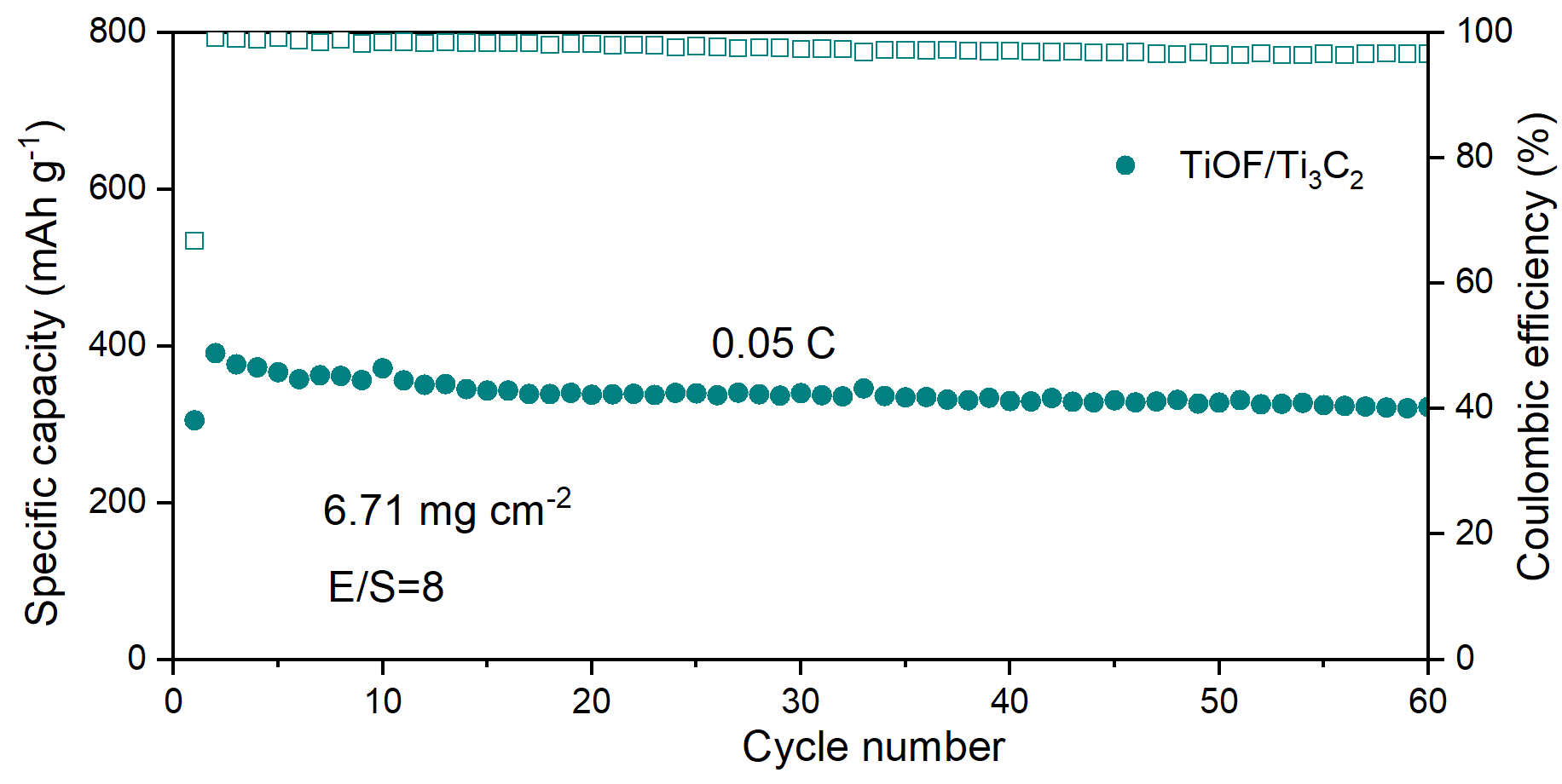


**Fig. S20** Cycling performance with a sulfur mass loading of 6.71 mg cm^-2^ at 0.05 C


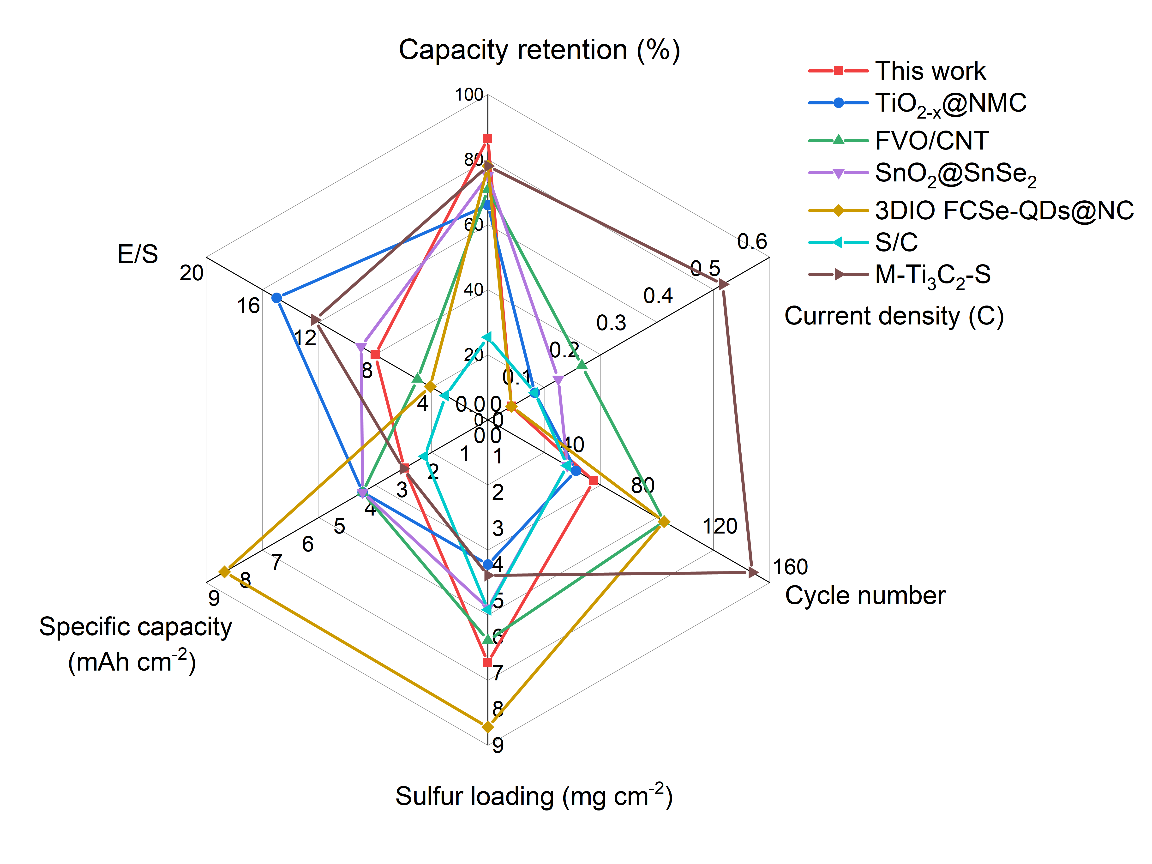


**Fig. S21** Li-S battery performance comparison

**Table S1** The element content of Ti_3_C_2_, TiO/Ti_3_C_2_, and TiOF/Ti_3_C_2_

| Materials | Ti (at%) | C (at%) | O (at%) | F (at%) |
| --- | --- | --- | --- | --- |
| TiOF/Ti_3_C_2_ | 24.06 | 44.96 | 23.63 | 7.36 |
| TiO/Ti_3_C_2_ | 21.85 | 40.99 | 32.13 | 5.03 |
| Ti_3_C_2_ | 22.19 | 36.76 | 25.89 | 14.25 |

**Table S2** Peaks potentials and potential difference from Fig. S10

| Electrode | Reduction peak (R1, R2)  (V) | Oxidation peak (O1, O2)  (V) | |
| --- | --- | --- | --- |
| Ti_3_C_2_ | 2.31, 2.037 | | 2.31, 2.378 |
| TiO/Ti_3_C_2_ | 2.33, 2.025 | | 2.295, 2.376 |
| TiOF/Ti_3_C_2_ | 2.306, 2.068 | | 2.308, 2.35 |

**Table S3** Bader charge of TiOF/Ti_3_C_2_, TiO/Ti_3_C_2_, and Ti_3_C_2_

|  | TiOF/Ti_3_C_2_ | TiO/Ti_3_C_2_ | Ti_3_C_2_ |
| --- | --- | --- | --- |
| Ti | -1.9409 | -1.1306 | -1.586 |

**Table S4** Shift values of binding energy for Li, F, S and Ti at different discharge/charge states

| Potentials | Li (eV) | F (eV) | S (eV) | Ti (eV) |
| --- | --- | --- | --- | --- |
| 2.14 V | 0 | 0 | 0 | 0 |
| 1.7 V | -0.2 | 0.6 | -1.09 | -0.3 |
| 2.42 V | 0 | 0.4 | 1.82 | 0 |
| 2.6 V | 0.2 | 0 | 2.13 | 0.5 |
